# MicroRNA Signatures in Lung Adenocarcinoma Metastases: Exploring the Oncogenic Targets of Tumor-Suppressive *miR-195-5p* and *miR-195-3p*

**DOI:** 10.3390/cancers17142348

**Published:** 2025-07-15

**Authors:** Yuya Tomioka, Naohiko Seki, Keiko Mizuno, Takayuki Suetsugu, Kentaro Tsuruzono, Yoko Hagihara, Mayuko Kato, Chikashi Minemura, Hajime Yonezawa, Kentaro Tanaka, Hiromasa Inoue

**Affiliations:** 1 Department of Pulmonary Medicine, Graduate School of Medical and Dental Sciences, Kagoshima University, Kagoshima 890-8520, Japan; k4829264@kadai.jp (Y.T.); keim@m.kufm.kagoshima-u.ac.jp (K.M.); taka3741@m2.kufm.kagoshima-u.ac.jp (T.S.); k1512489@kadai.jp (K.T.); k5382596@kadai.jp (Y.H.); k9288090@kadai.jp (K.T.); inoue@m2.kufm.kagoshima-u.ac.jp (H.I.); 2 Department of Functional Genomics, Graduate School of Medicine, Chiba University Chuo-ku, Chiba 260-8670, Japan; mayukokato@chiba-u.jp (M.K.); minemura@ndmc.ac.jp (C.M.); 3 Department of Neurosurgery, Graduate School of Medical and Dental Sciences, Kagoshima University, Kagoshima 890-8520, Japan; hajime@m3.kufm.kagoshima-u.ac.jp

**Keywords:** lung adenocarcinoma, microRNA, metastasis, expression signature, *miR-195-5p*, *miR-195-3p*, *ANLN*, *MAD2L1*

## Abstract

**Background**: To improve the prognosis of patients with lung adenocarcinoma (LUAD), revolutionary treatments for metastatic lesions are essential. **Methods**: To identify genes closely involved in LUAD-cell-derived metastasis, we used RNA sequencing to generate microRNA (miRNA) expression signatures of brain metastatic lesions. Once tumor-suppressive miRNAs are identified, it will be possible to explore the numerous tumor-promoting genes that are regulated by miRNAs. **Results**: By comparison with a previously created LUAD signature, we identified several miRNAs whose expression was significantly suppressed in brain metastases. We focused on both strands of pre-*miR-195* (*miR-195-5p* and *miR-195-3p*), which were significantly downregulated in brain metastatic tissues, and confirmed by ectopic expression assays that both strands of pre-*miR-195* attenuated the aggressive phenotypes (cell proliferation, migration, and invasion) of LUAD cells. These data suggest that both strands of pre-*miR-195* have tumor-suppressive functions in LUAD cells. Next, we explored the target molecules that each miRNA strand regulates in LUAD cells. We identified 159 target genes regulated by *miR-195-5p* and *miR-195-3p*, of which 12 genes (*ANLN*, *CDC6*, *CDCA2*, *CDK1*, *CEP55*, *CHEK1*, *CLSPN*, *GINS1*, *KIF23*, *MAD2L1*, *OIP5*, and *TIMELESS*) affect cell cycle/cell division and the prognosis of LUAD patients. Finally, we focused on two genes, *ANLN* (*miR-195-5p* target) and *MAD2L1* (*miR-195-3p* target), and demonstrated their oncogenic functions and the molecular pathways they regulate in LUAD cells. **Conclusions**: The miRNA signature derived from lung cancer brain metastasis will be a landmark in the field, and analysis of this miRNA signature will accelerate the identification of genes involved in lung cancer brain metastasis.

## Simple Summary

To identify genes involved in lung cancer brain metastasis, we generated microRNA signatures using lung cancer brain metastasis tissues. Based on the microRNA signatures in lung adenocarcinoma metastases, both strands of pre-*miR-195* (*miR-195-5p* and *miR-195-3p*) were significantly downregulated in metastatic tissues. A total of 12 genes (*ANLN*, *CDC6*, *CDCA2*, *CDK1*, *CEP55*, *CHEK1*, *CLSPN*, *GINS1*, *KIF23*, *MAD2L1*, *OIP5*, and *TIMELESS*) were identified as pre-*miR-195*-controlled genes, and these genes greatly contributed to the molecular pathogenesis of lung adenocarcinoma cells. Knockdown assays using siRNAs revealed that *ANLN* and *MAD2L1* facilitated the malignant transformation of lung adenocarcinoma cells. Analysis of the microRNA signature generated in this study will accelerate the identification of genes involved in lung cancer brain metastasis.

## 1. Introduction

Lung cancer is the most commonly diagnosed malignant tumor worldwide, with approximately 2.5 million new cases reported in 2022. It is also the leading cause of cancer-related deaths, accounting for 18.7% of cancer-related deaths worldwide [1]. Histologically, lung cancer is classified into non-small-cell lung cancer (NSCLC; which accounts for 85% of cases) and small-cell lung cancer (SCLC; which accounts for 15% of cases). Most NSCLC cases are adenocarcinoma (LUAD) [2]. In recent years, the prognosis of patients with advanced LUAD has improved with the advent of targeted therapies against specific genetic mutations and the introduction of immune checkpoint inhibitors [3,4]. However, despite these advances, distant metastasis remains the primary cause of lung-cancer-related deaths, with the brain being the most common site of dissemination [5,6]. Almost 10% of patients with newly diagnosed NSCLC present with metastasis, and 25–40% develop brain metastases during the disease course [6,7]. The prognosis for patients with brain metastases remains extremely poor, with the median survival ranging from only 3 to 6 months, even after treatment [8]. Therefore, our challenge is to elucidate the molecular mechanisms involved in the brain metastasis of lung cancer cells.

MicroRNAs (miRNAs) are small non-coding RNAs that act as post-transcriptional gene controllers and are essential for cell maintenance [9,10]. Numerous studies have demonstrated that aberrantly expressed miRNAs play important roles in the development, progression, metastasis, and drug resistance of cancer cells [11,12]. miRNA expression signatures provide a wealth of information about the miRNAs dysregulated in cancer cells [13]. Recent advances in RNA-sequencing technology have significantly contributed to the generation of miRNA expression signatures for various types of cancer cells [14]. Our research group has created miRNA expression signatures for various cancer tissues, including LUAD and SCLC, and identified tumor-suppressive miRNAs based on these signatures [15,16].

Our miRNA signatures demonstrated that some passenger strands of miRNAs were actually downregulated or upregulated in brain metastases.. Furthermore, we have demonstrated that some passenger strands, whose functions had previously been unclear, function as tumor-suppressive miRNAs in cancer cells and directly control multiple genes [15,16,17,18,19].

The aim of this study was to identify genes that are essential for lung cancer cells to metastasize to the brain. In this study, we generated a new miRNA expression signature from brain metastatic lesions and compared it with a signature from LUAD tissue that we had previously generated. Our signature showed that multiple passenger strands were downregulated in brain metastatic tissues. In particular, both the guide and passenger strands of the following eight pre-miRNAs were downregulated in metastatic tissues: *miR-10a*, *miR-34b*, *miR-34c*, *miR-195*, *miR-199a*, *miR-199b*, and *miR-497*. Exploring novel molecular pathways controlled by these miRNAs will provide new insights into the metastasis of LUAD cells. Interestingly, among these downregulated miRNAs, *miR-195* and *miR-497* are part of a cluster of miRNAs on human chromosome 17p13.1. The target genes of these clustered miRNAs may be involved in malignant transformation of LUAD cells.

To verify the validity of the new signature we created in this study, we focused on both strands of pre-*miR-195* (*miR-195-5p* and *miR-195-3p*), which were significantly downregulated in brain metastatic tissues, and attempted to search for their target molecules. Our functional assays confirmed that both strands of pre-*miR-195* acted as tumor suppressors in LUAD cells. A total of 159 genes were identified as target genes controlled by pre-*miR-195* in LUAD cells. We found that 12 of these genes (*ANLN*, *CDC6*, *CDCA2*, *CDK1*, *CEP55*, *CHEK1*, *CLSPN*, *GINS1*, *KIF23*, *MAD2L1*, *OIP5*, and *TIMELESS*) are involved in cell cycle/cell division and affect the prognosis of LUAD patients. Finally, we focused on two genes, *ANLN* (*miR-195-5p* target) and *MAD2L1 (miR-195-3p* target), and demonstrated their oncogenic functions and the molecular pathways regulated by them in LUAD cells.

## 2. Materials and Methods

### 2.1. LUAD Patients and LUAD Cell Lines

We obtained surgical specimens from the primary tumor and brain metastatic tissues of patients with LUAD. The background and clinical characteristics of the patients are described in Table S1.

We also employed two LUAD cell lines, A549 and H1299 (American Type Culture Collection, Manassas, VA, USA). The procedures for cell maintenance have been described in our previous publications [15,18].

### 2.2. Construction of an miRNA Expression Signature for LUAD Based on RNA Sequencing

To assess miRNA expression, LUAD and brain metastatic tissue samples were subjected to sequencing using the Illumina NextSeq 500 system (Illumina, Inc., San Diego, CA, USA). The raw sequencing data have been deposited in the Gene Expression Omnibus (GEO; GEO accession number: GSE230229).Sequence reads matched to the human genome were classified into different types of small RNAs based on their biological features, and the corresponding read counts for each category are presented in Table S2.

### 2.3. Identification of Oncogenic Targets of miR-195-5p and miR-195-3p in LUAD Cells

We analyzed gene expression data from A549 cells transfected with *miR-195-5p* or *miR-195-3p* (GEO accession number: GSE281258) in combination with predictions with TargetScanHuman ver. 8.0 (https://www.targetscan.org/vert_80/; accessed on 25 September 2024) to identify oncogenic targets regulated by *miR-195-5p* and *miR-195-3p*. This expression profile represents a comparison of the gene expression between LUAD and normal lung tissues. The molecular functions of the *miR-195-5p* and *miR-195-3p* target genes were inferred using GeneCodis4 software (https://genecodis.genyo.es/; accessed on 25 September 2024) [20].

### 2.4. Analysis of miRNA and Gene Expression and Their Clinical Significance in Patients with LUAD Using an In Silico Database

The expression of miRNAs and genes in LUAD clinical tissues was analyzed using the following publicly available databases: The Cancer Genome Atlas (TCGA) (https://www.cancer.gov/tcga; accessed on 20 September 2024), Genomic Data Commons Data Portal (https://portal.gdc.cancer.gov/; accessed on 20 September 2024), and FIREBROWSE (http://firebrowse.org/; accessed on 20 September 2024). Data related to overall survival were retrieved from OncoLnc (http://www.oncolnc.org/; downloaded on 20 September 2024) and cBioPortal (https://www.cbioportal.org/; accessed on 20 September 2024).

### 2.5. Functional Assays of miRNAs and miRNA Target Genes in LUAD Cells

miRNAs and small interfering RNAs (siRNAs) were transfected into LUAD cell lines. After transfection, cell proliferation, migration, and invasion were assessed and compared with those of untreated cells. The transfection procedures for the miRNAs and siRNAs were described in our previous studies [15,16,18]. The mock group comprised cells transfected without miRNAs or siRNAs. The cell cycle and apoptotic cells were analyzed using the BD FACSCelesta^TM^ Flow Cytometer (BD Biosciences, Franklin Lakes, NJ, USA). Details of the cell functional assays are described in our previous papers [15,16,18]. The reagents used for these analyses are listed in Table S3.

### 2.6. Dual-Luciferase Reporter Assay

The binding sequences of *miR-195-5p* and *miR-195-3p*, which were cloned into the psiCHEK2 vector (C8021; Promega, Madison, WI, USA), are shown in Figures S1 and S2. The procedures for the transfection and dual-luciferase reporter assays have been described in our previous studies [15,16,18]. We performed the dual-luciferase reporter assays at 72 h after transfection using the Dual-Luciferase Reporter Assay System (catalog no. E1910, Promega). The reagents used for these analyses are listed in Table S3.

### 2.7. Western Blotting

Western blotting was conducted following protocols described in our previous publications [15,16,18]. The reagents and antibodies used in the present study are listed in Table S3.

### 2.8. Statistical Analysis

Statistical analyses were performed using R ver. 4.4.0 (R Core Team, Vienna, Austria; https://www.R-project.org/; accessed on 25 September 2024) and GraphPad Prism 8 (GraphPad Software, La Jolla, CA, USA). Differences between two groups were assessed by the Student’s t-test. Multiple-group comparisons were performed using one-way analysis of variance and Tukey’s test for post hoc analysis. Survival analysis was carried out using Kaplan–Meier survival curves and the log-rank test.

## 3. Results

### 3.1. The RNA-Sequencing-Based miRNA Expression Signature Using Clinical Specimens Obtained from LUAD Brain Metastases

In this study, we created a new miRNA expression signature using LUAD brain metastatic tissues. We explored miRNAs whose expression was downregulated in brain metastasis tissues by comparing them with the LUAD signature we had previously created [15]. A total of 48 downregulated miRNAs were identified (Table 1), and 14 miRNAs were annotated as passenger strands based on the miRBase database (Release 22; http://www.mirbase.org/) [21]. Interestingly, both the guide and the passenger strands derived from the following pre-miRNAs were significantly downregulated: *miR-10a*, *miR-34b*, *miR-34c*, *miR-195*, *miR-199a*, *miR-199b*, and *miR-497* (Table 1). Exploring the genes controlled by these miRNAs will provide novel information regarding brain metastasis of LUAD cells.

**Figure 1:**
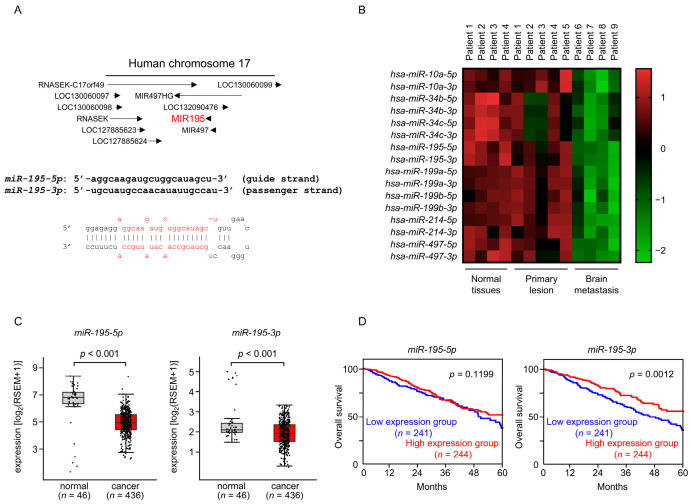
Expression patterns of *miR-195-5p* and *miR-195-3p* in LUAD clinical specimens and their association with 5-year overall survival rates. (**A**) Genomic location of pre-*miR-195* on the human chromosome. The mature sequences of *miR-195-5p* (guide strand) and *miR-195-3p* (passenger strand) are indicated. (**B**) Heatmap of the expression levels of miRNAs downregulated in brain metastases across normal lung tissue, LUAD tissue, and brain metastases, based on the LUAD miRNA signature obtained by RNA sequencing. (**C**) Expression analysis of *miR-195-5p* and *miR-195-3p* using the TCGA-LUAD dataset. (**D**) Kaplan–Meier survival curves showing the 5-year overall survival of LUAD patients (*n* = 485), stratified by the median expression level of *miR-195-5p* or *miR-195-3p*. High-and low-expression groups are shown in red and blue, respectively.

In the human genome, *miR-195* and *miR-497* are part of a cluster of miRNAs on human chromosome 17p13.1 (Figure 1A). Both *miR-195* and *miR-497* were significantly downregulated in brain metastatic tissues compared with LUAD and normal lung tissues (Figure 1B). Analysis of the functional RNA network controlled by this miRNA cluster could potentially elucidate the molecular mechanism of lung cancer cell metastasis. Here, we first focused on *miR-195-5p* and *miR-195-3p* to identify their regulated RNA networks in LUAD cells.

### 3.2. Expression and Clinical Significance of miR-195-5p and miR-195-3p in LUAD Clinical Specimens

We validated the expression of *miR-195-5p* and *miR-195-3p* in LUAD clinical specimens using the large clinical cohort database TCGA. The expression levels of *miR-195-5p* and *miR-195-3p* were significantly reduced in LUAD tissues compared with normal tissues (Figure 1C). Using the TCGA dataset, we analyzed whether the expression levels of these miRNAs can serve as prognostic markers for LUAD. Low expression of *miR-195-3p* was associated with a significantly poor prognosis compared with high expression of this miRNA (Figure 1D). A similar analysis showed no significant difference in the expression of *miR-195-5p* among the tissue types (Figure 1D).

### 3.3. Tumor-Suppressive Functions of miR-195-5p and miR-195-3p in LUAD Cells

**Figure 2:**
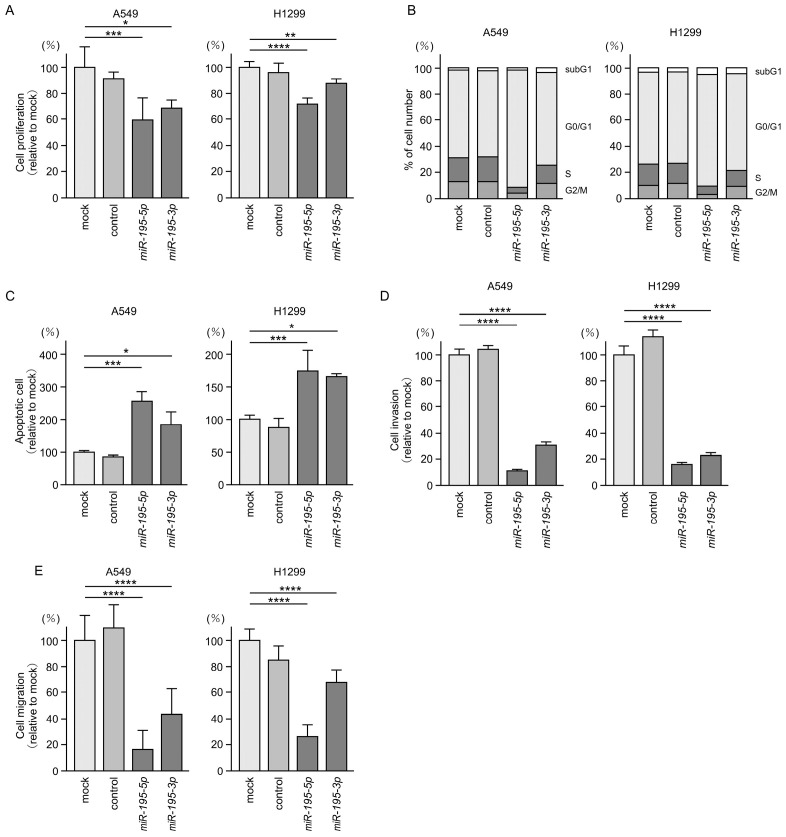
Antitumor effects of *miR-195-5p* and *miR-195-3p* in LUAD cells (A549 and H1299). (**A**) Cell proliferation evaluated using XTT assays 72 h after transient transfection with *miR-195-5p* or *miR-195-3p*. (**B**) Cell cycle status analyzed by flow cytometry 72 h after transient transfection with *miR-195-5p* or *miR-195-3p*. (**C**) Apoptotic cells evaluated by flow cytometry following Annexin V-FITC and PI-PerCP-Cy5-5-A staining 72 h after transient transfection with *miR-195-5p* or *miR-195-3p.* (**D**) Cell invasion assessed using Matrigel invasion assays. Cells were transfected with *miR-195-5p* or *miR-195-3p* for 72 h prior to seeding. (**E**) Cell migration assessed using a membrane culture system. Cells were transfected with *miR-195-5p* or *miR-195-3p* for 72 h prior to seeding. *, *p* < 0.05; **, *p* < 0.01; ***, *p* < 0.001; ****, *p* < 0.0001.

To demonstrate the antitumor functions of both strands of pre-*miR-195* in LUAD cells, we ectopically expressed these miRNAs in A549 and H1299 cell lines and examined their effects on cancer cell behavior. Ectopic expression of *miR-195-5p* or *miR-195-3p* significantly suppressed the proliferation of LUAD cells (Figure 2A). Cell cycle analysis revealed the typical G0/G1 phase arrest following transfection with either miRNA (Figure 2B). Furthermore, expression of *miR-195-5p* or *miR-195-3p* led to a marked increase in the apoptotic cell population (Figure 2C). In addition, the invasive and migratory capacities of LUAD cells were significantly inhibited upon ectopic expression of *miR-195-5p* or *miR-195-3p* (Figure 2D,E). Flow cytometry images demonstrating this apoptosis are presented in Figure S3, and representative images of the invasion and migration assays are shown in Figure S4. Based on these results, we conclude that *miR-195-5p* and *miR-195-3p* function as tumor-suppressive miRNAs in LUAD cells.

### 3.4. Identification of miR-195-5p- and miR-195-3p-Regulated Genes in LUAD Cells

**Figure 3:**
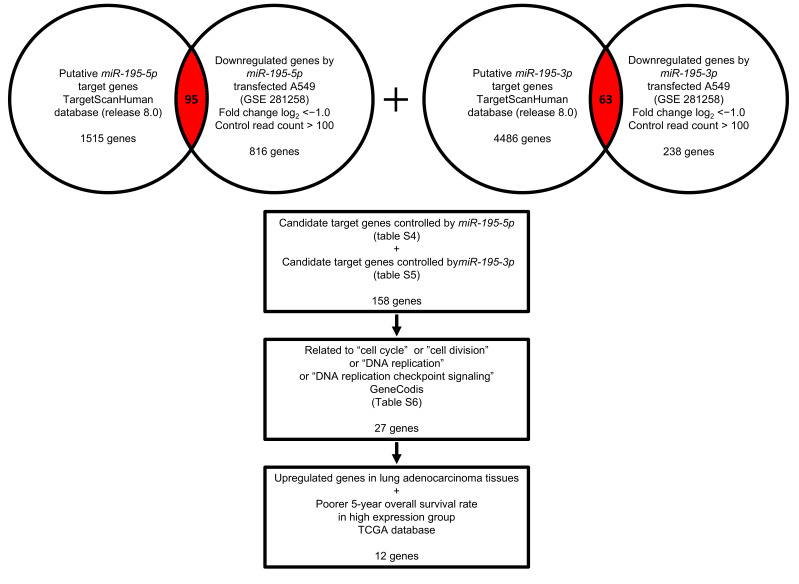
Strategy for identifying oncogenic targets regulated by *miR-195-5p* or *miR-195-3p* in LUAD cells. Two datasets were utilized to identify the target genes: the TargetScanHuman database (release 8.0) and our original mRNA expression dataset (GEO accession number: GSE281258). Associations with LUAD patient prognosis were evaluated using two databases: OncoLnc and cBioPortal.

As reported previously, we confirmed that both strands of pre-*miR-195* function as antitumor miRNAs. Identifying the oncogenic targets regulated by these miRNAs in LUAD cells will aid in the search for genes involved in the malignant progression of lung cancer. Our strategy for the microRNA target search is shown in Figure 3. In this study, we performed a genome-wide gene expression analysis in A549 cells transfected with *miR-195-5p* or *miR-195-3p*. The expression data obtained have been deposited in the GEO database and are accessible (accession number GSE281258).

Using a combination of the TargetScanHuman database (release 8.0) and the gene expression profiles obtained from miRNA-transfected LUAD cells, we identified putative target genes regulated by *miR-195-5p* (95 genes) and *miR-195-3p* (63 genes). These candidate targets are listed in Tables S4 and S5. To further characterize their potential roles, we analyzed the molecular functions of all 158 genes using the GeneCodis4 database (Table S6). Notably, 27 of these genes were associated with cell cycle regulation (Table 2).

### 3.5. Clinical Significance of Genes Regulated by miR-195-5p or miR-195-3p in LUAD

**Figure 4:**
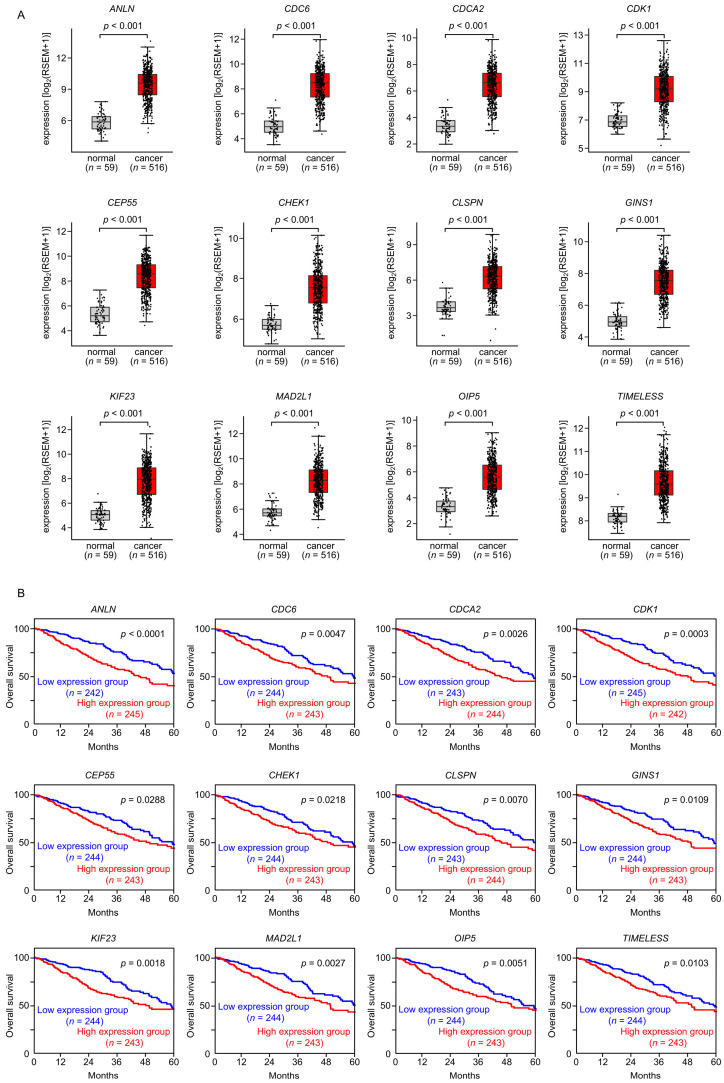
Expression levels of the 12 target genes regulated by *miR-195-5p* or *miR-195-3p* in LUAD cells, along with their associated 5-year overall survival rates. (**A**) The expression levels of the 12 target genes of *miR-195-5p* or *miR-195-3p* (*ANLN*, *CDC6*, *CDCA2*, *CDK1*, *CEP55*, *CHEK1*, *CLSPN*, *GINS1*, *KIF23*, *MAD2L1*, *OIP5*, and *TIMELESS*) in LUAD clinical specimens assessed using the TCGA-LUAD dataset. (**B**) Kaplan–Meier curves for the 5-year overall survival rate based on the expression of the 12 target genes (*ANLN*, *CDC6*, *CDCA2*, *CDK1*, *CEP55*, *CHEK1*, *CLSPN*, *GINS1*, *KIF23*, *MAD2L1*, *OIP5*, and *TIMELESS*). The patients (*n* = 487) were divided into high- and low-expression groups based on the median expression level of each gene. The red and blue lines denote the high- and low-expression groups, respectively.

**Figure 5:**
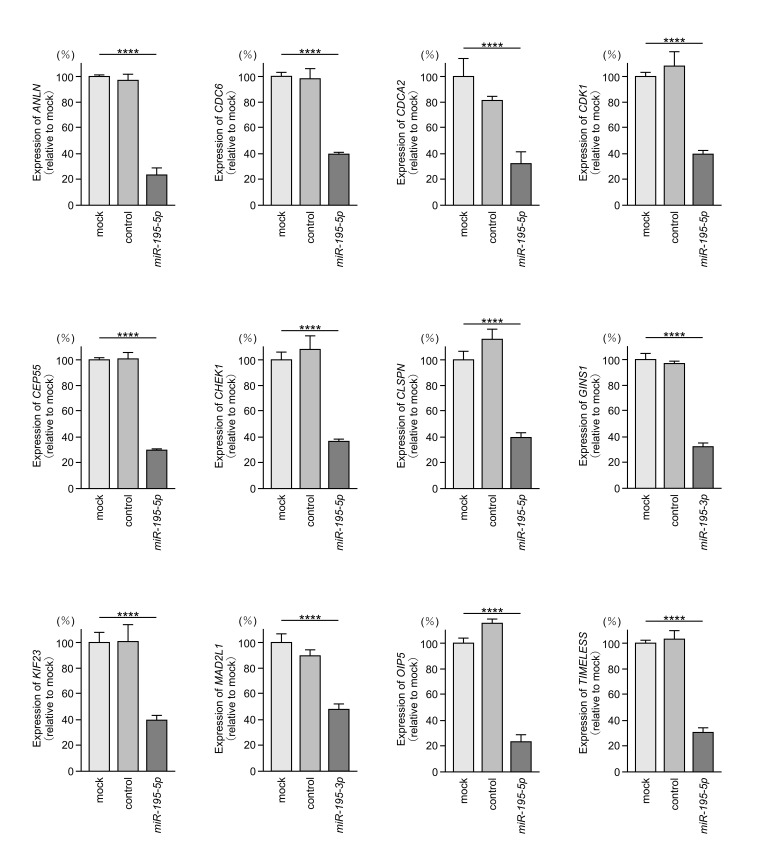
Regulation of the 12 target genes by *miR-195-5p* or *miR-195-3p* in A549 cells. Total RNA was extracted 72 h after miRNA transfection and analyzed by quantitative real-time PCR. *GAPDH* was used as an internal control. ****, *p* < 0.0001.

To assess the clinical relevance of the 27 genes potentially regulated by *miR-195-5p* or *miR-195-3p*, we analyzed the TCGA-LUAD dataset. Among the 27 target genes, 12 (Table 2, bold) were significantly upregulated in LUAD tissues (*n* = 516) compared with normal lung tissues (*n* = 59) (Figure 4A). Furthermore, elevated expression of these genes was significantly associated with a poor prognosis (5-year overall survival rate, *p* < 0.05) in LUAD patients (Figure 4B). Ectopic expression of *miR-195-5p* or *miR-195-3p* in LUAD cells significantly reduced the mRNA levels of these 12 target genes (Figure 5). Among the 12 target genes, we focused on anillin (*ANLN*), the top prognostic candidate regulated by *miR-195-5p*, and mitotic arrest deficient 2-like 1 (*MAD2L1*), the top prognostic candidate regulated by *miR-195-3p*, for further functional analyses.

### 3.6. Direct Regulation of ANLN by miR-195-5p and MAD2L1 by miR-195-3p in LUAD Cells

**Figure 6:**
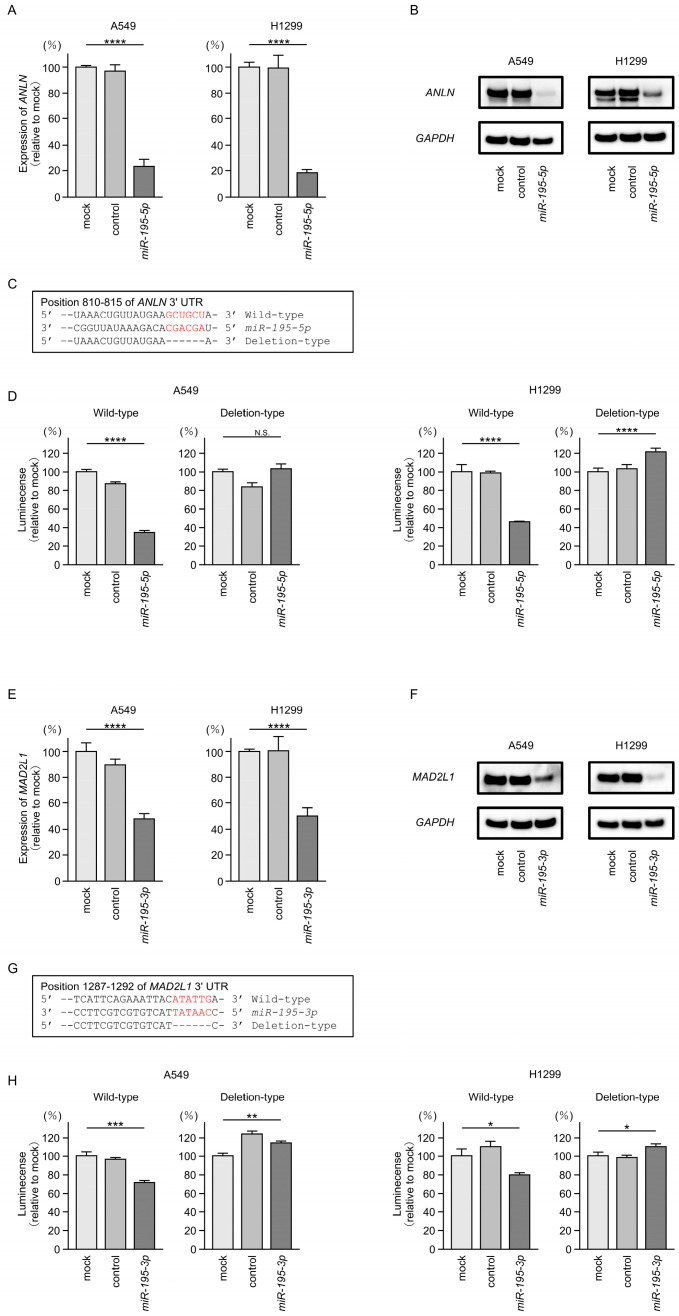
Direct regulation of *ANLN* and *MAD2L1* by *miR-195* expression in LUAD cells. (**A**,**E**) Marked reduction in *ANLN* or *MAD2L1* mRNA expression by ectopic expression of *miR-195-5p* or *miR-195-3p* in LUAD cells (A549 and H1299). Total RNA was isolated 72 h after miRNA transfection and quantified by real-time PCR. *GAPDH* was used as an internal control. (**B**,**F**) Significant reduction in ANLN or MAD2L1 protein levels by ectopic expression of *miR-195-5p* or *miR-195-3p* in LUAD cells (A549 and H1299). Protein samples were isolated 72 h after *miR-195-5p* or *miR-195-3p* transfection and quantified by Western blotting. GAPDH was used as an internal control. (**C**,**G**) Putative *miR-195-5p-* or *miR-195-3p*-binding sites in the 3′-UTR of *ANLN* or *MAD2L1*, detected using the TargetScanHuman database (release 8.0). (**D**,**H**) Dual-luciferase reporter assays revealing the reduced luminescence activity after co-transfection of *miR-195-5p* or *miR-195-3p* with a vector containing the *miR-195-5p-* or *miR-195-3p*-binding site (wild-type) in LUAD cells (A549 and H1299). *, *p* < 0.05; **, *p* < 0.01; ***, *p* < 0.001; ****, *p* < 0.0001; N.S., not significant.

Ectopic expression of *miR-195-5p* and *miR-195-3p* in LUAD cells significantly reduced the mRNA and protein expression levels of ANLN and MAD2L1, respectively (Figure 6A,B,E,F). Full-length Western blot images are provided in Figures S5 and S6. Luciferase reporter assays further demonstrated that both miRNAs directly bind to the 3′ untranslated regions (3′-UTRs) of their respective target genes. The predicted *miR-195-5p*-binding site in the *ANLN* 3′-UTR and the *miR-195-3p*-binding site in the *MAD2L1* 3′-UTR are shown in Figure 6C and G, respectively. Co-transfection of *miR-195-5p* or *miR-195-3p* with the corresponding reporter construct led to a marked reduction in luciferase activity, whereas no such reduction was observed after transfection with constructs lacking the respective binding sites (Figure 6D,H). These findings indicate that *miR-195-5p* and *miR-195-3p* directly target *ANLN* and *MAD2L1*, respectively, and post-transcriptionally suppress their expression in LUAD cells.

### 3.7. Functional Significance of ANLN in LUAD Cells

**Figure 7:**
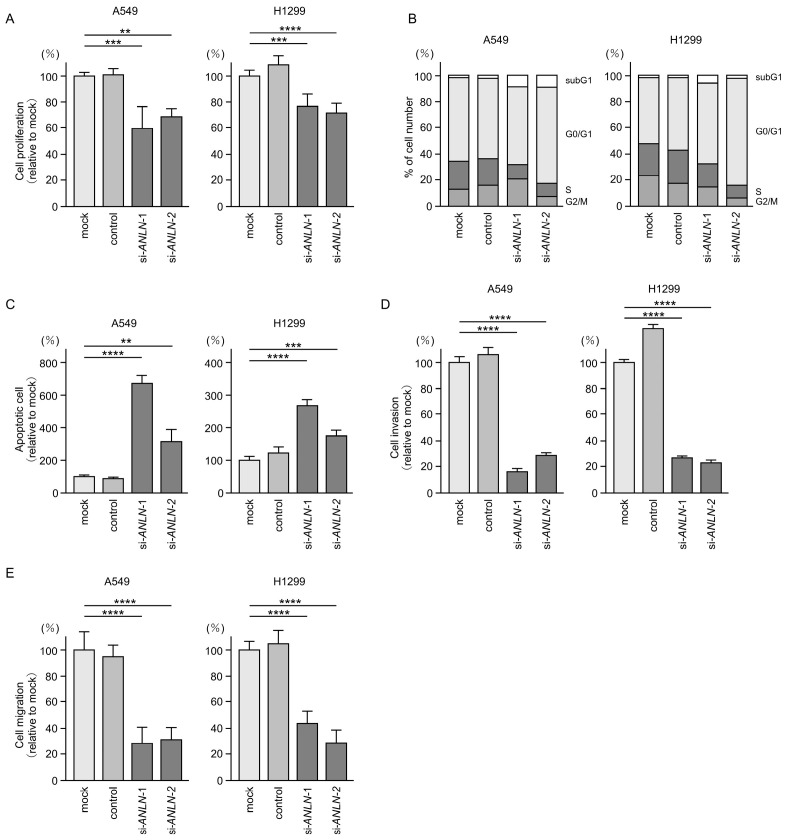
Effects of *ANLN* knockdown by siRNAs in LUAD cells (A549 and H1299). (**A**) Cell proliferation evaluated using XTT assays after transient transfection with si*ANLN*-1 or si*ANLN*-2. (**B**) Cell cycle status analyzed by flow cytometry 72 h after transient transfection with si*ANLN*-1 or si*ANLN*-2. (**C**) Apoptotic cells evaluated by flow cytometry following Annexin V-FITC and PI-PerCP-Cy5-5-A staining 72 h after transient transfection with si*ANLN*-1 or si*ANLN*-2. (**D**) Cell invasion assessed using Matrigel invasion assays. Cells were transfected with si*ANLN*-1 or si*ANLN*-2 for 72 h prior to seeding. (**E**) Cell migration assessed using a membrane culture system. Cells were transfected with si*ANLN*-1 or si*ANLN*-2 for 72 h prior to seeding. **, *p* < 0.01; ***, *p* < 0.001; ****, *p* < 0.0001.

To investigate the oncogenic role of *ANLN* in LUAD cells, we performed siRNA-mediated knockdown assays. Transfection with two independent siRNAs targeting *ANLN* (si*ANLN*-1 and si*ANLN*-2) resulted in significant reductions in both *ANLN* mRNA and protein levels in LUAD cells (Figures S7 and S8). Cell proliferation assays revealed that *ANLN* knockdown inhibited the proliferation of LUAD cells (Figure 7A). Furthermore, flow cytometric analysis showed that *ANLN* knockdown in LUAD cells induced G0/G1 cell cycle arrest and increased the proportion of apoptotic cells (Figure 7B,C). Notably, *ANLN* knockdown markedly suppressed both the invasive and migratory capacities of LUAD cells (Figure 7D,E). Flow cytometry images showing the proportion of cells in apoptosis are presented in Figure S9, and representative images of the invasion and migration assays are shown in Figure S10.

### 3.8. Functional Significance of MAD2L1 in LUAD Cells

**Figure 8:**
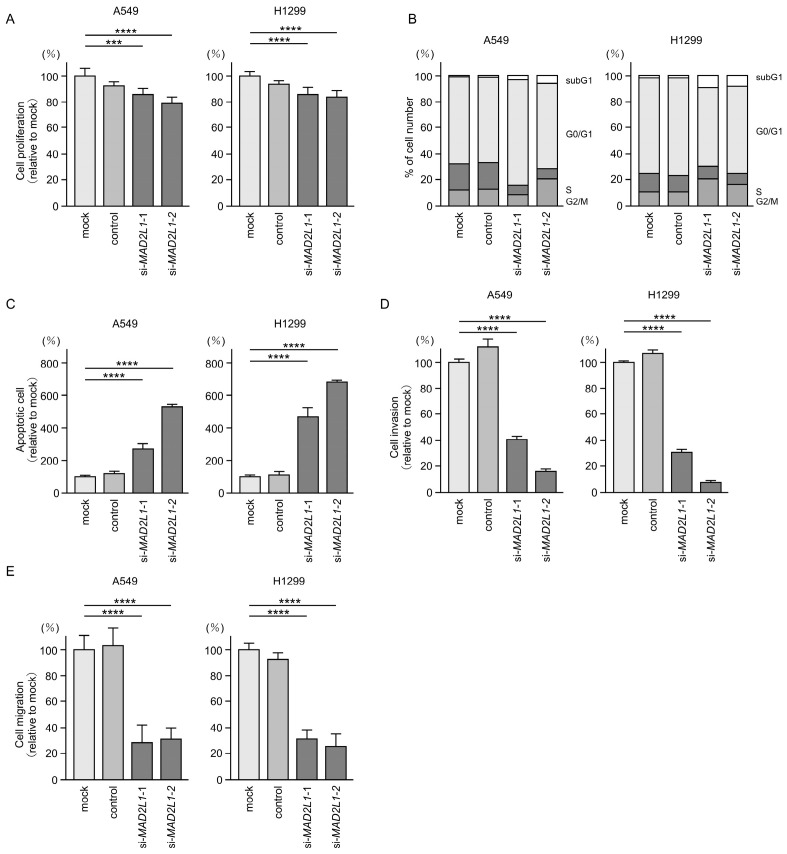
Effects of *MAD2L1* knockdown by siRNAs in LUAD cells (A549 and H1299). (**A**) Cell proliferation evaluated using XTT assays after transient transfection with si*MAD2L1*-1 or si*MAD2L1*-2. (**B**) Cell cycle status analyzed by flow cytometry 72 h after transient transfection with si*MAD2L1*-1 or si*MAD2L1*-2. (**C**) Apoptotic cells evaluated by flow cytometry following Annexin V-FITC and PI-PerCP-Cy5-5-A staining 72 h after transient transfection with si*MAD2L1*-1 or si*MAD2L1*-2. (**D**) Cell invasion assessed using Matrigel invasion assays. Cells were transfected with si*MAD2L1*-1 or si*MAD2L1*-2 for 72 h prior to seeding. (**E**) Cell migration assessed using a membrane culture system. Cells were transfected with si*MAD2L1*-1 or si*MAD2L1*-2 for 72 h prior to seeding. ***, *p* < 0.001; ****, *p* < 0.0001.

Similarly, to investigate the oncogenic role of *MAD2L1* in LUAD cells, we performed siRNA-mediated knockdown assays. Transfection with two independent siRNAs targeting *MAD2L1* (si*MAD2L1*-1 and si*MAD2L1*-2) resulted in significant reductions in both *MAD2L1* mRNA and protein expression levels in LUAD cells (Figures S11 and S12). Cell proliferation assays showed that *MAD2L1* knockdown slightly inhibited the proliferation of LUAD cells (Figure 8A). Additionally, flow cytometric analysis demonstrated that *MAD2L1* knockdown induced G0/G1 cell cycle arrest and increased the proportion of apoptotic cells in LUAD cells (Figure 8B,C). However, in H1299 cells, no increase in the proportion of G0/G1 phase cells was observed; instead, there was a notable increase in the proportion of subG1 cells, indicating enhanced apoptotic activity. Furthermore, *MAD2L1* knockdown markedly suppressed the invasive and migratory abilities of LUAD cells (Figure 8D,E). Flow cytometry images showing the proportion of cells in apoptosis are presented in Figure S13, and representative images of the invasion and migration assays are shown in Figure S14.

### 3.9. ANLN- and MAD2L1-Mediated Cancer Pathways in LUAD Cells

To investigate the signaling pathways influenced by *ANLN* and *MAD2L1* in cancer, we generated genome-wide gene expression profiles using A549 cells transfected with si*ANLN* or si*MAD2L1*. The resultant expression data have been deposited in the GEO database under accession numbers GSE281585 and GSE281905.

**Figure 9:**
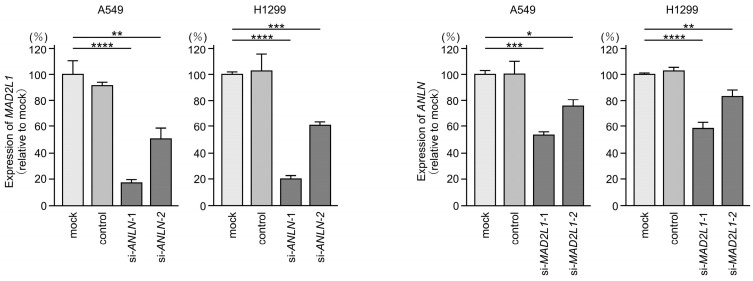
Reciprocal suppression of *ANLN* and *MAD2L1* mRNA levels by siRNA transfection in A549 and H1299 cells. Total RNA was extracted 72 h after siRNA transfection and analyzed by quantitative real-time PCR. *GAPDH* was used as an internal control. *, *p* < 0.05; **, *p* < 0.01; ***, *p* < 0.001; ****, *p* < 0.0001.

**Figure 10:**
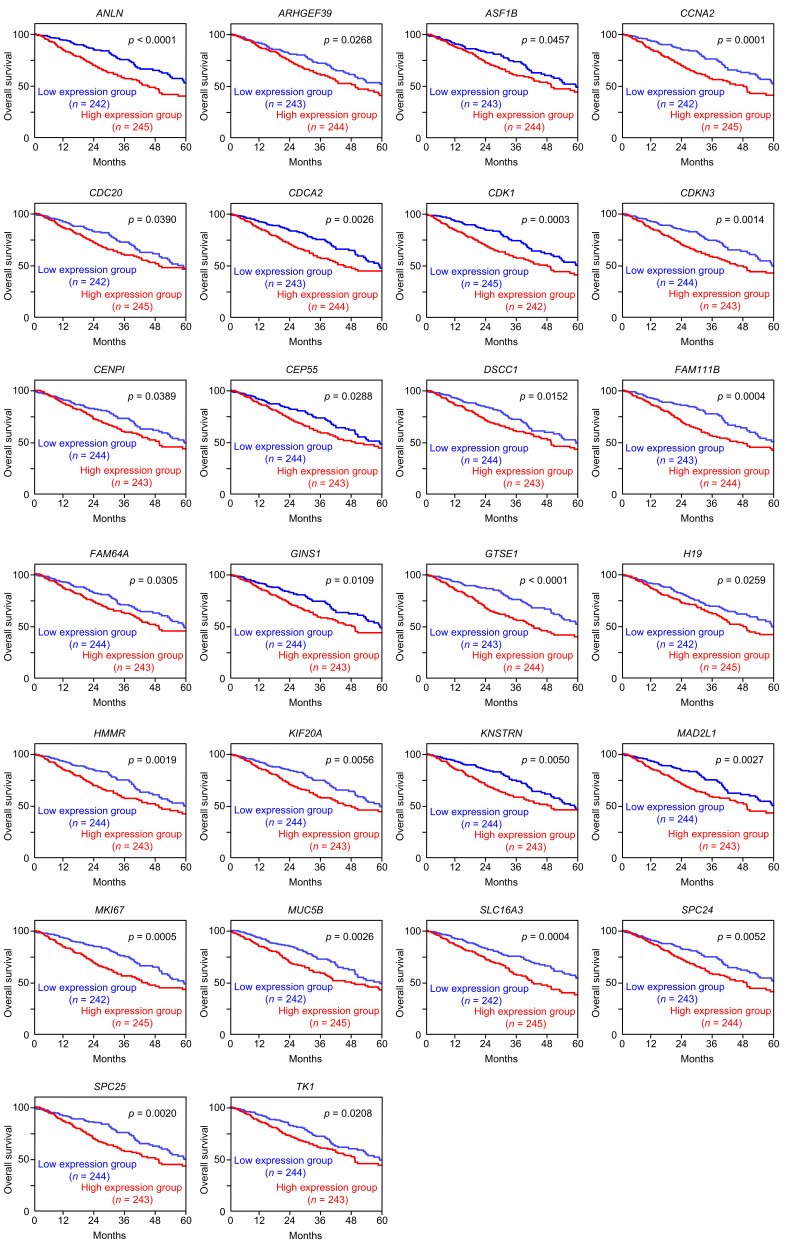
Kaplan–Meier survival curves showing the 5-year overall survival based on the expression of the 26 genes negatively associated with the LUAD patient prognosis. The patients (*n* = 487) were stratified by the median expression levels of each target gene. High- and low-expression groups are shown in red and blue, respectively.

Interestingly, we also observed that si*ANLN* transfection suppressed *MAD2L1* expression, while si*MAD2L1* transfection suppressed *ANLN* expression, suggesting the existence of reciprocal regulatory interactions between these two genes (Figure 9). A total of 39 genes were commonly downregulated in both si*ANLN*- and si*MAD2L1*-transfected cells (Table 3). These genes were analyzed using GeneCodis4, which revealed that a substantial proportion of the genes are involved in cell-cycle-related pathways (Table 4). Among these genes, the expression of 26 genes had a negative effect on the prognosis of LUAD patients (Figure 10).

## 4. Discussion

In this study, we focused on both strands of pre-*miR-195* (*miR-195-5p* and *miR-195-3p*), which were significantly downregulated in brain metastatic tissues. Furthermore, we explored the target molecules that each miRNA controls in LUAD cells. A total of 159 genes were identified as target genes controlled by pre-*miR-195*. We found that 12 of these genes (*ANLN*, *CDC6*, *CDCA2*, *CDK1*, *CEP55*, *CHEK1*, *CLSPN*, *GINS1*, *KIF23*, *MAD2L1*, *OIP5*, and *TIMELESS*) are involved in cell cycle/cell division and affect the prognosis of LUAD patients. Using in vitro functional assays, we revealed that *ANLN* and *MAD2L1* actually play an oncogenic role.

Our group has previously identified tumor-suppressive miRNAs and their novel regulatory RNA networks in lung cancer cells [15,18,19]. RNA-sequencing-based miRNA signatures provide important information that suggests which miRNAs should be analyzed. Our recent studies revealed that passenger strands derived from pre-miRNAs function as oncogenes or tumor suppressors and actually regulate intracellular target molecules in LUAD cells [18,19]. Similarly to previous results, this study also showed that both the passenger strand and guide strand of *miR-195* equally and essentially affect the function of cancer cells (Figure 2). Comprehensive analysis of the passenger strands as well as the guide strands of miRNAs will bring new information to cancer research.

An interesting feature of this signature was that it contained several miRNAs (e.g., *miR-10a*, *miR-34b*, *miR-34c*, *miR-195*, *miR-199a*, *miR-199b*, and *miR-497*) in which both strands (guide and passenger strands derived from the pre-miRNAs) were significantly downregulated in brain metastatic tissues. Analysis of these miRNAs, including their passenger strands, will shed light on the RNA networks associated with lung cancer metastasis. Among these miRNAs, we focused on the *miR-195*/*miR-497* miRNA cluster on human chromosome 17p13.1. We first investigated the tumor-suppressive functions of *miR-195* and its regulated RNA networks in LUAD cells.

Previous reports have shown that the expression of *miR-195-5p* (the guide strand) is suppressed in various types of cancers. Furthermore, ectopic expression assays demonstrated that *miR-195-5p* acts as a tumor-suppressive miRNA across cancer types by targeting several oncogenes [22,23,24,25]. Expression of *miR-195-5p* enhanced the cisplatin or gemcitabine sensitivity of LUAD cells by targeting E2F transcription factor 7 [26]. Recent studies have demonstrated that aberrantly expressed long non-coding RNAs or circulating RNAs may be involved in promoting cancer cell malignant transformation by adsorbing tumor-suppressive *miR-195-5p* in LUAD cells [27]. Compared with the numerous studies on *miR-195-5p*, functional assays of *miR-195-3p* (the passenger strand) are scarce. Our present analysis demonstrates that *miR-195-3p* is a tumor-suppressive miRNA that regulates many cancer genes in LUAD cells. Combining our data with previous reports, it is clear that both strands of pre-*miR-195* (*miR-195-5p* and *miR-195-3p*) behave as tumor suppressors in LUAD cells.

Next, we identified cancer-promoting genes among those regulated by pre-*miR-195* (*miR-195-5p* and *miR-195-3p*) in LUAD cells. Our strategy successfully identified 159 genes as candidate pre-*miR-195* targets in LUAD cells. Of these, 12 genes (*ANLN*, *CDC6*, *CDCA2*, *CDK1*, *CEP55*, *CHEK1*, *CLSPN*, *GINS1*, *KIF23*, *MAD2L1*, *OIP5*, and *TIMELESS*) are involved in the cell cycle, cell division, and DNA replication checkpoint-related pathways, and their overexpression has a significant impact on the prognosis of patients with LUAD; therefore, these genes are potential therapeutic targets in LUAD. Among these therapeutic targets, we focused on *ANLN* (*miR-195-5p* target) and *MAD2L1* (*miR-195-3p* target) and revealed their cancer-promoting functions in LUAD cells.

*ANLN* is an actin-binding protein that interacts with actin and cytoskeletal filaments to control the cell cycle [28]. Recent studies show that aberrant expression of *ANLN* is observed in multiple types of cancer, and that overexpression of *ANLN* is a useful biomarker predicting cancer cell metastasis and patient prognosis [29]. Knockdown assays targeting ANLN expression revealed dramatically reduced aggressive phenotypes (cell growth, migration, and invasion) of cancer cells, including pancreatic, breast, and lung cancers [30,31,32]. Aberrant expression of *ANLN* is intricately involved in the molecular pathogenesis of various human cancers and is a potential therapeutic target.

*MAD2L1* is an essential component of the spindle assembly checkpoint [33]. In normal cells, *MAD2* accumulates at kinetochores before cell division to ensure that spindle microtubules are properly aligned with the kinetochores of each chromosome [33]. Thus, *MAD2* is a gatekeeper of cell cycle checkpoints and is essential for maintaining faithful replicative cell division and preventing cancer development [34]. In multiple cancers, overexpression of *MAD2L1* is involved in the malignant transformation of cancer cells, and patients overexpressing *MAD2L1* have a poor prognosis [35,36,37,38]. More recently, it has been reported that *MAD2L1* expression is involved in carboplatin resistance in lung cancer cells [39]. *MAD2L1* serves as a therapeutic target molecule in various cancers, including LUAD.

Examination of the interaction between *ANLN* and *MAD2L1* showed that these two genes affect each other. In addition, the existence of genes whose expression is commonly regulated downstream of *ANLN* and *MAD2L1* was revealed. The expression of 26 of these genes had a negative effect on the prognosis of LUAD patients. Our study has shown that suppression of *miR-195-5p*/*-3p* expression and overexpression of the *ANLN*/*MAD2L1* genes are among the factors that accelerate the progression of lung cancer cells. Future studies involving in vivo validation or analyses using larger clinical datasets, including independent patient cohorts, will be essential to confirm the biological and therapeutic significance of the identified targets.

Analyses based on the miRNA signature created in this study will facilitate the identification of genes involved in the malignant progression and brain metastasis of LUAD cells. This study is exploratory and based on a limited number of LUAD brain metastasis specimens, which are rare and difficult to obtain. While the findings offer important insights, they should be interpreted with caution and require further validation in larger patient cohorts to confirm their broader applicability.

## 5. Conclusions

We generated a novel RNA-sequencing-based miRNA signature using clinical specimens obtained from lung cancer brain metastases. Both strands of pre-*miR-195* (*miR-195-5p* and *miR-195-3p*) were significantly downregulated in brain tissues. Ectopic expression assays demonstrated that the two miRNAs derived from pre-*miR-195* attenuated LUAD cell aggressiveness by targeting several oncogenes, particularly *ANLN* (*miR-195-5p* target) and *MAD2L1* (*miR-195-3p* target). *ANLN* and *MAD2L1* may be novel therapeutic targets in LUAD cells.

## Figures and Tables

**Table 1:** Downregulated miRNAs in brain metastasis compared with those in lung adenocarcinoma, identified by RNA sequencing.

MicroRNA	miRBase Accession No.	Guide or Passenger Strand	Chromosomal Location	Log_2_ FC	*p*-Value	FDR
*hsa-miR-30d-5p*	MIMAT0000245	Guide strand	8q24.22	−2.51	0.004	0.018
*hsa-miR-34b-5p*	MIMAT0000685	Passenger strand	11q23.1	−2.56	0.047	0.117
*hsa-miR-4536-5p*	MIMAT0019078	Guide strand	Xp11.21	−2.56	0.001	0.005
*hsa-miR-6785-3p*	MIMAT0027471	Passenger strand	17q25.1	−2.59	0.000	0.000
*hsa-miR-3141*	MIMAT0015010	Guide strand	5q33.2	−2.61	0.008	0.031
*hsa-miR-30b-5p*	MIMAT0000420	Guide strand	8q24.22	−2.63	0.003	0.016
*hsa-miR-497-3p*	MIMAT0004768	Passenger strand	17p13.1	−2.63	0.000	0.003
*hsa-miR-4322*	MIMAT0016873	Guide strand	19p13.2	−2.64	0.016	0.053
*hsa-miR-195-3p*	MIMAT0004615	Passenger strand	17p13.1	−2.66	0.000	0.001
*hsa-miR-6743-3p*	MIMAT0027388	Guide strand	11p15.5	−2.66	0.000	0.002
*hsa-miR-148a-5p*	MIMAT0004549	Passenger strand	7p15.2	−2.79	0.000	0.000
*hsa-miR-195-5p*	MIMAT0000461	Guide strand	17p13.1	−2.79	0.000	0.002
*hsa-miR-548i*	MIMAT0005935	Guide strand	3q21.2	−2.81	0.008	0.032
*hsa-miR-6788-3p*	MIMAT0027477	Guide strand	18p11.22	−2.82	0.000	0.000
*hsa-miR-363-5p*	MIMAT0003385	Passenger strand	Xq26.2	−2.85	0.001	0.005
*hsa-miR-4536-3p*	MIMAT0020959	Passenger strand	Xp11.21	−2.91	0.000	0.000
*hsa-miR-497-5p*	MIMAT0002820	Guide strand	17p13.1	−2.91	0.000	0.002
*hsa-miR-214-5p*	MIMAT0004564	Passenger strand	1q24.3	−2.92	0.000	0.001
*hsa-miR-95-3p*	MIMAT0000094	Guide strand	4p16.1	−2.92	0.008	0.033
*hsa-miR-4662a-5p*	MIMAT0019731	Guide strand	8q24.13	−2.94	0.008	0.031
*hsa-miR-145-5p*	MIMAT0000437	Guide strand	5q32	−2.95	0.000	0.002
*hsa-miR-130a-3p*	MIMAT0000425	Guide strand	11q12.1	−3.02	0.000	0.002
*hsa-miR-504-5p*	MIMAT0002875	Guide strand	Xq26.3	−3.02	0.012	0.042
*hsa-miR-199a-3p*	MIMAT0000232	Guide strand	19p13.2	−3.03	0.000	0.002
*hsa-miR-34c-3p*	MIMAT0004677	Passenger strand	11q23.1	−3.06	0.009	0.036
*hsa-miR-6763-5p*	MIMAT0027426	Guide strand	12q24.33	−3.07	0.000	0.000
*hsa-miR-214-3p*	MIMAT0000271	Guide strand	1q24.3	−3.14	0.000	0.002
*hsa-miR-199b-3p*	MIMAT0004563	Guide strand	9q34.11	−3.15	0.000	0.002
*hsa-miR-218-5p*	MIMAT0000275	Guide strand	4p15.31	−3.17	0.001	0.006
*hsa-miR-34c-5p*	MIMAT0000686	Guide strand	11q23.1	−3.22	0.042	0.107
*hsa-miR-4470*	MIMAT0018997	Guide strand	8q12.3	−3.30	0.000	0.001
*hsa-miR-1281*	MIMAT0005939	Guide strand	22q13.2	−3.32	0.000	0.000
*hsa-miR-4259*	MIMAT0016880	Guide strand	1q23.2	−3.33	0.000	0.000
*hsa-miR-3064-3p*	MIMAT0019865	Passenger strand	17q23.3	−3.41	0.000	0.000
*hsa-miR-3926*	MIMAT0018201	Guide strand	8p23.1	−3.44	0.000	0.000
*hsa-miR-5091*	MIMAT0021083	Guide strand	4p15.33	−3.46	0.000	0.001
*hsa-miR-199b-5p*	MIMAT0000263	Passenger strand	9q34.11	−3.56	0.001	0.007
*hsa-miR-602*	MIMAT0003270	Guide strand	9q34.3	−3.61	0.000	0.003
*hsa-miR-548ar-3p*	MIMAT0022266	Passenger strand	13q34	−3.62	0.000	0.000
*hsa-miR-10a-3p*	MIMAT0004555	Passenger strand	17q21.32	−3.82	0.001	0.004
*hsa-miR-10a-5p*	MIMAT0000253	Guide strand	17q21.32	−3.82	0.000	0.002
*hsa-miR-548w*	MIMAT0015060	Guide strand	16p12.1	−3.85	0.000	0.000
*hsa-miR-199a-5p*	MIMAT0000231	Passenger strand	19p13.2	−3.91	0.000	0.000
*hsa-miR-150-5p*	MIMAT0000451	Guide strand	19q13.33	−4.00	0.000	0.001
*hsa-miR-548a*	MIMAT0003251	Guide strand	6p22.3	−4.19	0.000	0.000
*hsa-miR-34b-3p*	MIMAT0004676	Guide strand	11q23.1	−4.32	0.005	0.023

**Table 2:** Putative target genes regulated by *miR-195-5p* or *miR-195-3p* in A549 cells.

Gene ID	Gene Symbol	Gene Name	Downregulated by *miR-195-5p* or *miR-195-3p*	*miR-195-5p * Transfectant Log_2_ Fold Change	*miR-195-3p* Transfectant Log_2_ Fold Change
402	*ARL2*	ADP ribosylation factor-like GTPase 2	*miR-195-5p*	−3.31	0.11
896	*CCND3*	cyclin D3	*miR-195-5p*	−3.00	0.74
11339	***OIP5***	Opa-interacting protein 5	*miR-195-5p*	−2.49	−0.62
54443	***ANLN***	anillin, actin-binding protein	*miR-195-5p*	−2.35	−0.36
55165	***CEP55***	centrosomal protein 55	*miR-195-5p*	−2.27	−0.67
983	***CDK1***	cyclin-dependent kinase 1	*miR-195-5p*	−1.86	−0.63
63967	***CLSPN***	claspin	*miR-195-5p*	−1.85	−0.70
993	*CDC25A*	cell division cycle 25A	*miR-195-5p*	−1.80	0.18
990	***CDC6***	cell division cycle 6	*miR-195-5p*	−1.78	−0.54
157313	***CDCA2***	cell division cycle- associated 2	*miR-195-5p*	−1.78	−0.53
55038	*CDCA4*	cell division cycle- associated 4	*miR-195-5p*	−1.75	−0.28
1111	***CHEK1***	checkpoint kinase 1	*miR-195-5p*	−1.56	−0.31
6867	*TACC1*	transforming acidic coiled-coil-containing protein 1	*miR-195-5p*	−1.52	0.27
9493	***KIF23***	kinesin family member 23	*miR-195-5p*	−1.52	−0.11
80010	*RMI1*	RecQ-mediated genome instability 1	*miR-195-3p*	−1.52	−1.07
144455	*E2F7*	E2F transcription factor 7	*miR-195-5p*	−1.46	−0.01
4085	***MAD2L1***	mitotic arrest deficient 2-like 1	*miR-195-3p*	−1.44	−2.13
8914	***TIMELESS***	timeless circadian clock	*miR-195-5p*	−1.39	−0.23
79187	*FSD1*	fibronectin type III and SPRY domain-containing 1	*miR-195-5p*	−1.26	−0.72
6197	*RPS6KA3*	ribosomal protein S6 kinase A3	*miR-195-5p*	−1.24	0.62
27183	*VPS4A*	vacuolar protein sorting 4 homolog A	*miR-195-5p*	−1.16	−0.07
9874	*TLK1*	tousled-like kinase 1	*miR-195-5p*	−1.12	0.13
9837	***GINS1***	GINS complex subunit 1	*miR-195-3p*	−1.08	−1.85
996	*CDC27*	cell division cycle 27	*miR-195-5p*	−1.06	−0.11
8243	*SMC1A*	structural maintenance of chromosomes 1A	*miR-195-3p*	−0.47	−1.32
7329	*UBE2I*	ubiquitin-conjugating enzyme E2 I	*miR-195-3p*	−0.03	−1.00
57804	*POLD4*	DNA polymerase delta 4	*miR-195-3p*	0.19	−1.35

**Table 3:** Downregulated genes after si*ANLN*-1 or si*MAD2L1*-1 transfection in A549 cells.

Entrez Gene ID	Gene Symbol	Gene Name	si*ANLN* Transfectant Log_2_ Fold Change	si*MAD2L1* Transfectant Log_2_ Fold Change
54443	*ANLN*	anillin actin-binding protein	−6.14	−2.32
84904	*ARHGEF39*	Rho guanine nucleotide exchange factor 39	−3.61	−2.28
55723	*ASF1B*	anti-silencing function 1B histone chaperone	−4.52	−2.71
890	*CCNA2*	cyclin A2	−3.64	−2.09
991	*CDC20*	cell division cycle 20	−4.26	−2.67
157313	*CDCA2*	cell division cycle-associated 2	−3.01	−2.03
55536	*CDCA7L*	cell division cycle-associated 7-like	−1.55	−2.61
983	*CDK1*	cyclin-dependent kinase 1	−4.60	−1.96
1033	*CDKN3*	cyclin-dependent kinase inhibitor 3	−2.92	−2.43
2491	*CENPI*	centromere protein I	−3.73	−2.60
55165	*CEP55*	centrosomal protein 55	−3.59	−2.03
1164	*CKS2*	CDC28 protein kinase regulatory subunit 2	−2.68	−1.55
1719	*DHFR*	dihydrofolate reductase	−4.01	−2.18
79075	*DSCC1*	DNA replication and sister chromatid cohesion 1	−2.76	−2.39
374393	*FAM111B*	family with sequence similarity 111 member B	−4.26	−3.13
54478	*FAM64A*	family with sequence similarity 64 member A	−3.89	−1.92
9837	*GINS1*	GINS complex subunit 1	−2.36	−3.05
51512	*GTSE1*	G2 and S-phase-expressed 1	−4.21	−2.36
283120	*H19*	H19, imprinted maternally expressed transcript	−2.58	−2.98
8479	*HIRIP3*	HIRA-interacting protein 3	−2.21	−1.93
3161	*HMMR*	hyaluronan-mediated motility receptor	−3.78	−2.60
55806	*HR*	hair growth-associated	−1.94	−1.91
56992	*KIF15*	kinesin family member 15	−4.13	−2.51
10112	*KIF20A*	kinesin family member 20A	−3.86	−2.05
90417	*KNSTRN*	kinetochore localized astrin/SPAG5-binding protein	−1.65	−1.25
4085	*MAD2L1*	MAD2 mitotic arrest deficient-like 1	−2.91	−5.58
4288	*MKI67*	marker of proliferation Ki-67	−4.47	−3.08
727897	*MUC5B*	mucin 5B, oligomeric mucus/gel-forming	−1.82	−1.00
23397	*NCAPH*	non-SMC condensin I complex subunit H	−3.76	−2.26
9768	*PCLAF*	PCNA clamp-associated factor	−4.43	−2.75
5933	*RBL1*	RB transcriptional corepressor like 1	−1.87	−2.54
10535	*RNASEH2A*	ribonuclease H2 subunit A	−2.93	−2.08
200916	*RPL22L1*	ribosomal protein L22 like 1	−1.07	−2.64
9123	*SLC16A3*	solute carrier family 16 member 3	−1.13	−1.57
147841	*SPC24*	SPC24, NDC80 kinetochore complex component	−3.75	−2.40
57405	*SPC25*	SPC25, NDC80 kinetochore complex component	−4.89	−2.89
7083	*TK1*	thymidine kinase 1	−4.42	−2.45
8458	*TTF2*	transcription termination factor 2	−1.57	−1.46
89891	*WDR34*	WD repeat domain 34	−1.21	−1.80

**Table 4:** Significantly enriched annotations of downregulated genes after si*ANLN*-1 or si*MAD2L1*-1 transfection.

Description	*p*-Value	Genes
cell division	<0.001	*MAD2L1, CDC20, NCAPH, CCNA2, ANLN, SPC25, FAM64A, KNSTRN, CDCA2, CEP55, CDK1, SPC24, CDCA7L, CKS2*
DNA replication	<0.001	*FAM111B, RNASEH2A, KIAA0101, GINS1, DSCC1, CDK1*
mitotic spindle assembly checkpoint signaling	<0.001	*MAD2L1, CDC20, SPC25, SPC24*
chromosome segregation	<0.001	*SPC25, KNSTRN, CDCA2, SPC24, CENPI*
mitotic cell cycle phase transition	<0.001	*CCNA2, CDK1, CKS2*
mitotic sister chromatid segregation	<0.001	*MAD2L1, KNSTRN, CENPI*
regulation of chromosome segregation	<0.001	*MKI67, CDCA2*
mitotic cytokinesis	<0.001	*KIF20A, ANLN, CEP55*
G1/S transition in the mitotic cell cycle	<0.001	*CCNA2, CDK1, CDKN3*
mitotic sister chromatid cohesion	<0.001	*CDC20, DSCC1*

## Data Availability

Publicly available datasets were analyzed in this study. These data can be accessed here: https://www.ncbi.nlm.nih.gov/geo/query/acc.cgi?acc=GSE230229 (accessed on 25 September 2024), https://www.ncbi.nlm.nih.gov/geo/query/acc.cgi?acc=GSE281258 (accessed on 24 November 2024) and https://www.ncbi.nlm.nih.gov/geo/query/acc.cgi?acc=GSE281905 (accessed on 24 November 2024).

## Supplementary Materials

The following supporting information can be downloaded at: https://www.mdpi.com/article/10.3390/cancers17142348/s1, Figure S1: Predicted *miR-195-5p* binding site in *ANLN* and inserted sequences in the luciferase vectors; Figure S2: Predicted *miR-195-3p* binding site in *MAD2L1* and inserted sequences in the luciferase vectors; Figure S3: Flow cytometry plots of Annexin V/PI-stained cells showing apoptosis after *miR-195-5p* or *miR-195-3p* transfection in A549 and H1299 cells; Figure S4: Representative images of invasion and migration assays after *miR-195-5p* or *miR-195-3p* transfection in A549 and H1299 cells; Figure S5: Full-sized Western blot images of ANLN expression after ectopic *miR-195-5p* expression in A549 and H1299 cells; Figure S6: Full-sized Western blot images of MAD2L1 expression after ectopic *miR-195-3p* expression in A549 and H1299 cells; Figure S7: Suppression of *ANLN* mRNA levels by si*ANLN*-1 or si*ANLN*-2 transfection in A549 and H1299 cells; Figure S8: Full-sized Western blot images showing ANLN expression after si*ANLN*-1 or si*ANLN*-2 transfection in A549 and H1299 cells; Figure S9: Flow cytometry plots of Annexin V/PI-stained cells showing apoptosis after si*ANLN*-1 or si*ANLN*-2 transfection in A549 and H1299 cells; Figure S10: Representative images of invasion and migration assays after si*ANLN*-1 or si*ANLN*-2 transfection in A549 and H1299 cells; Figure S11: Suppression of *MAD2L1* mRNA levels by si*MAD2L1*-1 or si*MAD2L1*-2 transfection in A549 and H1299 cells; Figure S12: Full-sized Western blot images showing MAD2L1 expression after si*MAD2L1*-1 or si*MAD2L1*-2 transfection in A549 and H1299 cells; Figure S13: Flow cytometry plots of Annexin V/PI-stained cells showing apoptosis after si*MAD2L1*-1 or si*MAD2L1*-2 transfection in A549 and H1299 cells; Figure S14: Representative images of invasion and migration assays after si*MAD2L1*-1 or si*MAD2L1*-2 transfection in A549 and H1299 cells; Table S1: Clinical features of the LUAD patients who provided tissues at the time of initial diagnosis; Table S2: Annotations of reads aligned to small RNAs; Table S3: Reagents used in this study; Table S4: Putative target genes regulated by *miR-195-5p* in A549 cells; Table S5: Putative target genes regulated by *miR-195-3p* in A549 cells, Table S6: Significantly enriched annotations of target genes regulated by *miR-195-5p* or *miR-195-3p*.
