# Supplementary figures and images for: MicroRNA Signatures in Lung Adenocarcinoma Metastases: Exploring the Oncogenic Targets of Tumor-Suppressive miR-195-5p and miR-195-3p
Source: Cancers (Basel). 2025 Jul 15;17(14):2348. doi: 10.3390/cancers17142348 (PMC12293978; doi:10.3390/cancers17142348)

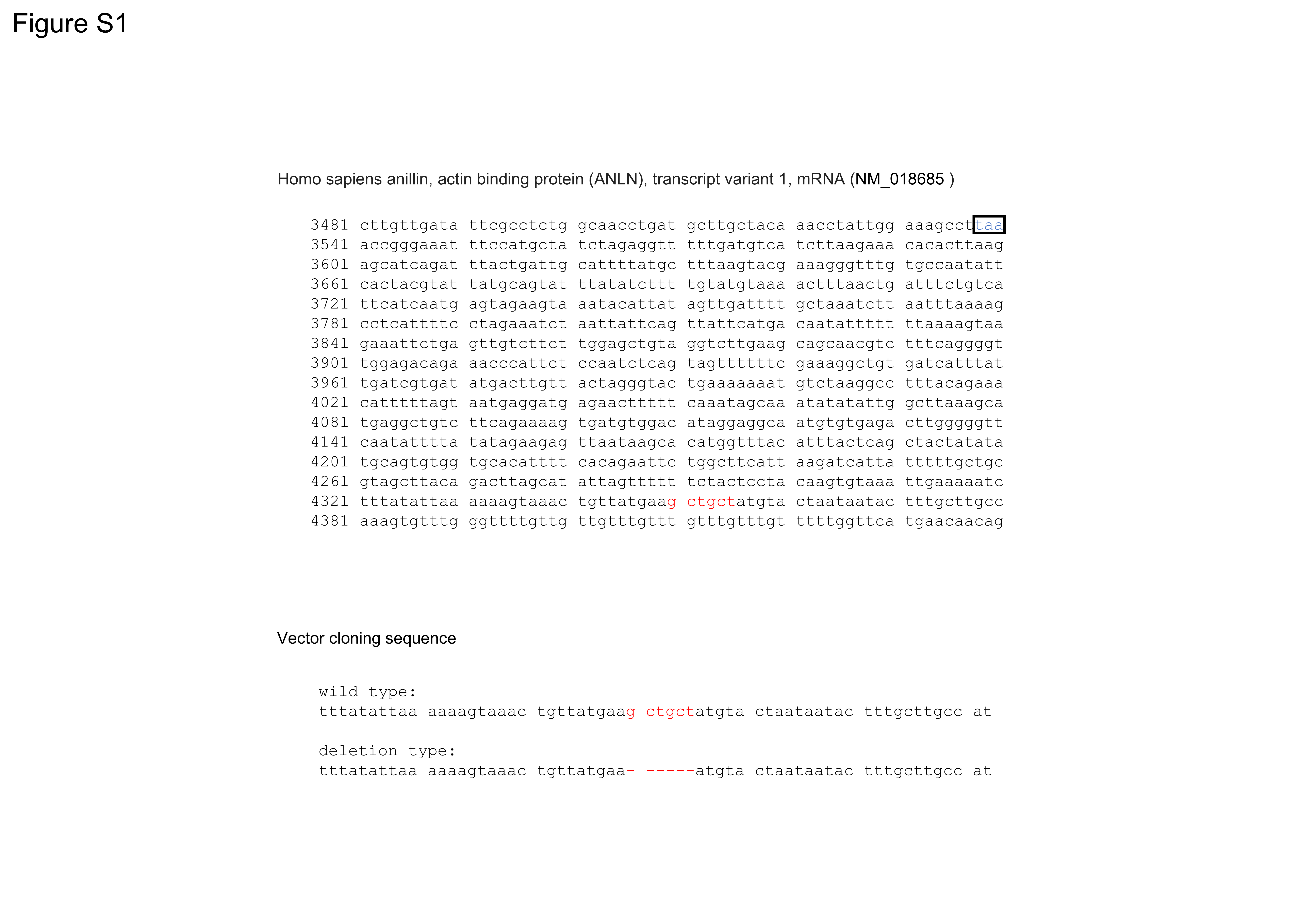

Supplement: Supplementary file 1 [file cancers-17-02348-s001.zip › cancers-3723267/Figure S1.tif]

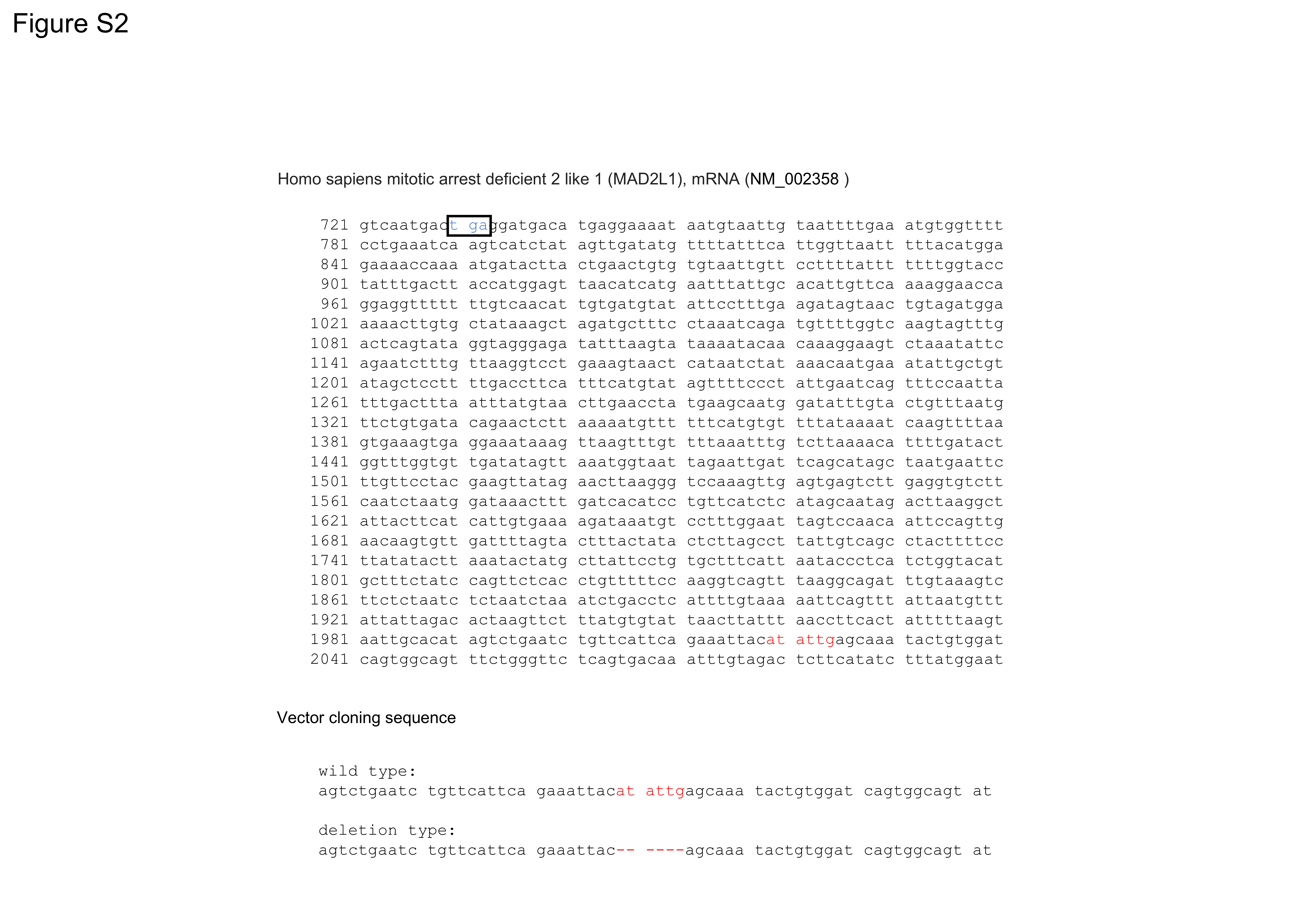

Supplement: Supplementary file 1 [file cancers-17-02348-s001.zip › cancers-3723267/Figure S2.tif]

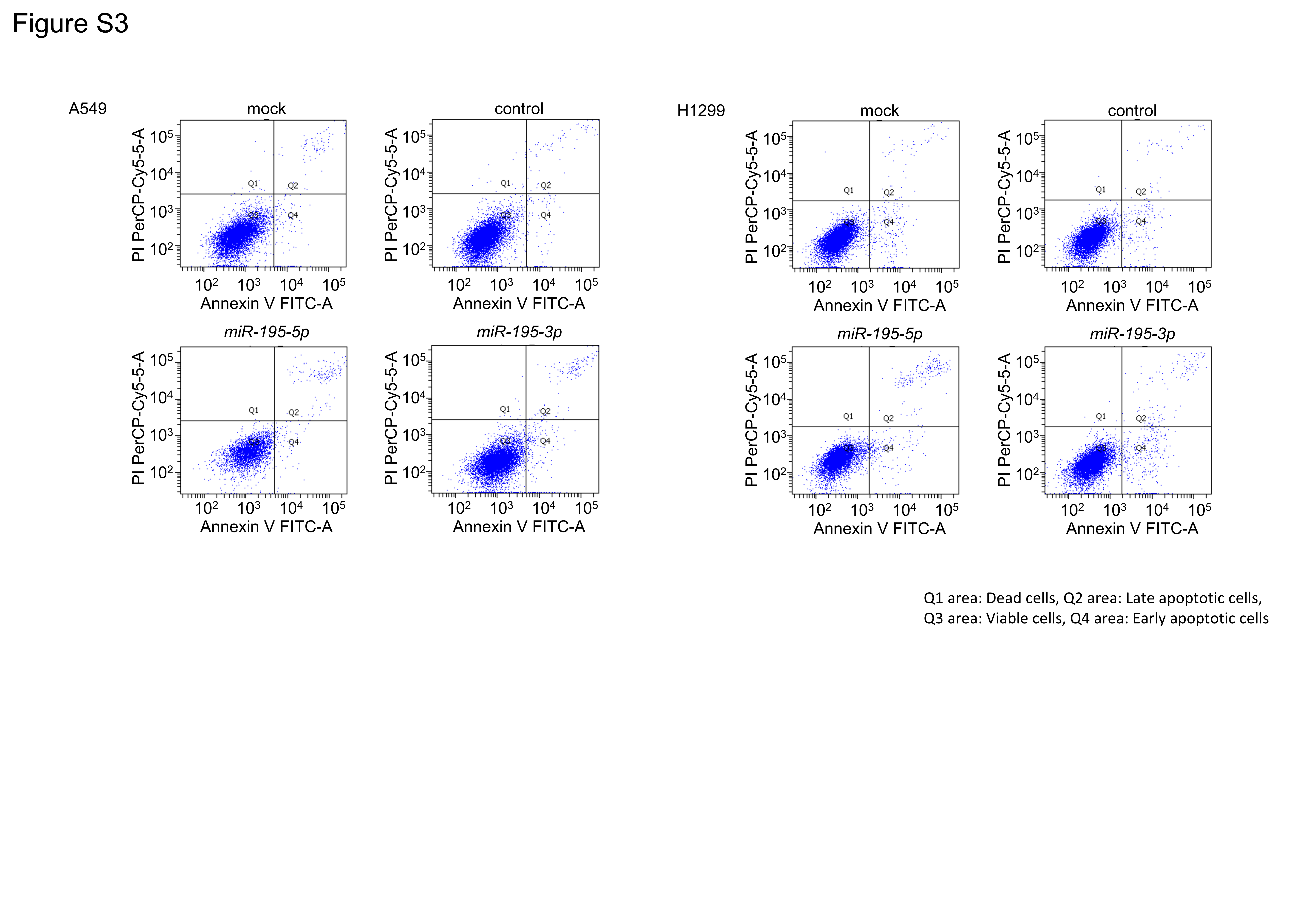

Supplement: Supplementary file 1 [file cancers-17-02348-s001.zip › cancers-3723267/Figure S3.tif]

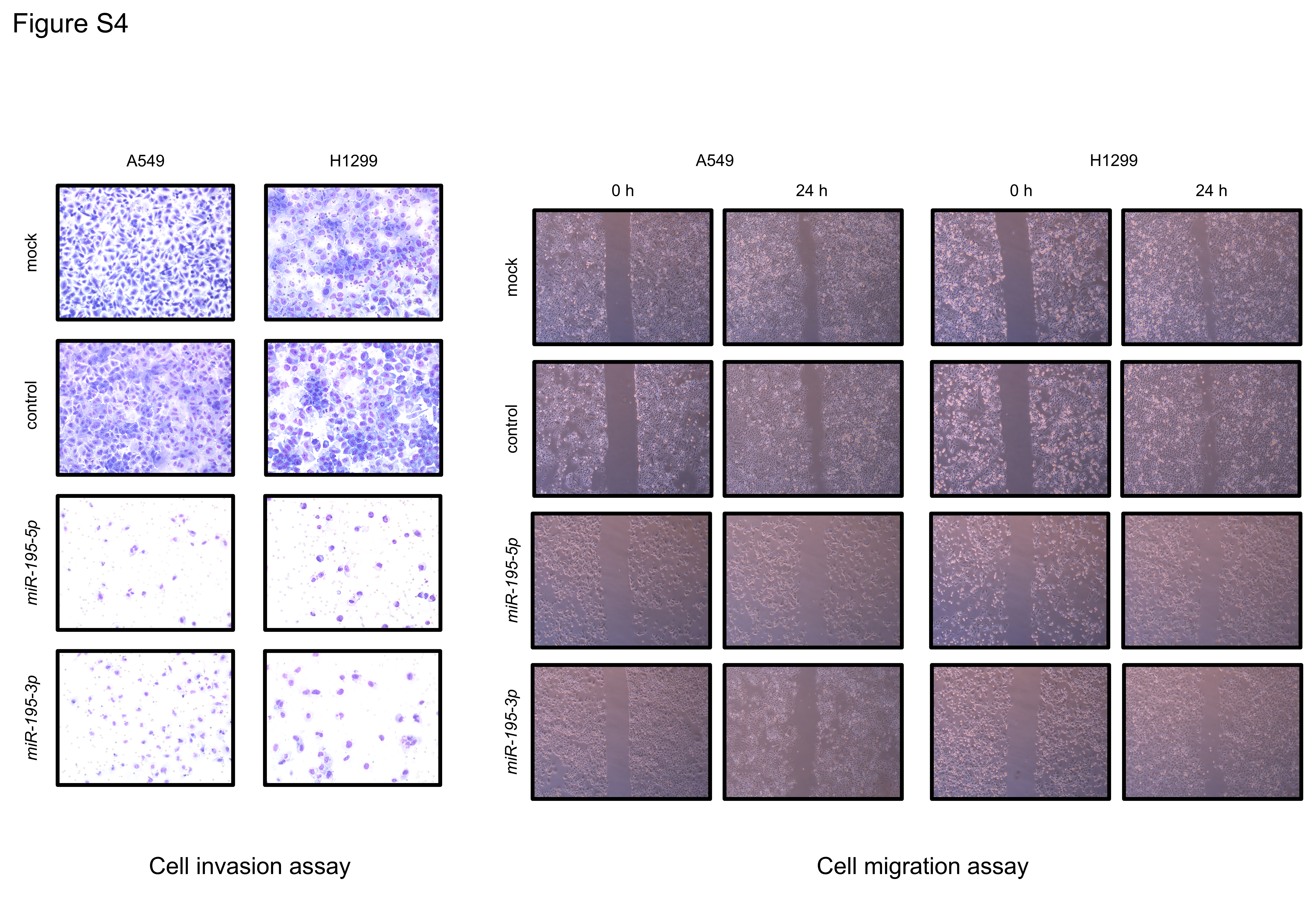

Supplement: Supplementary file 1 [file cancers-17-02348-s001.zip › cancers-3723267/Figure S4.tif]

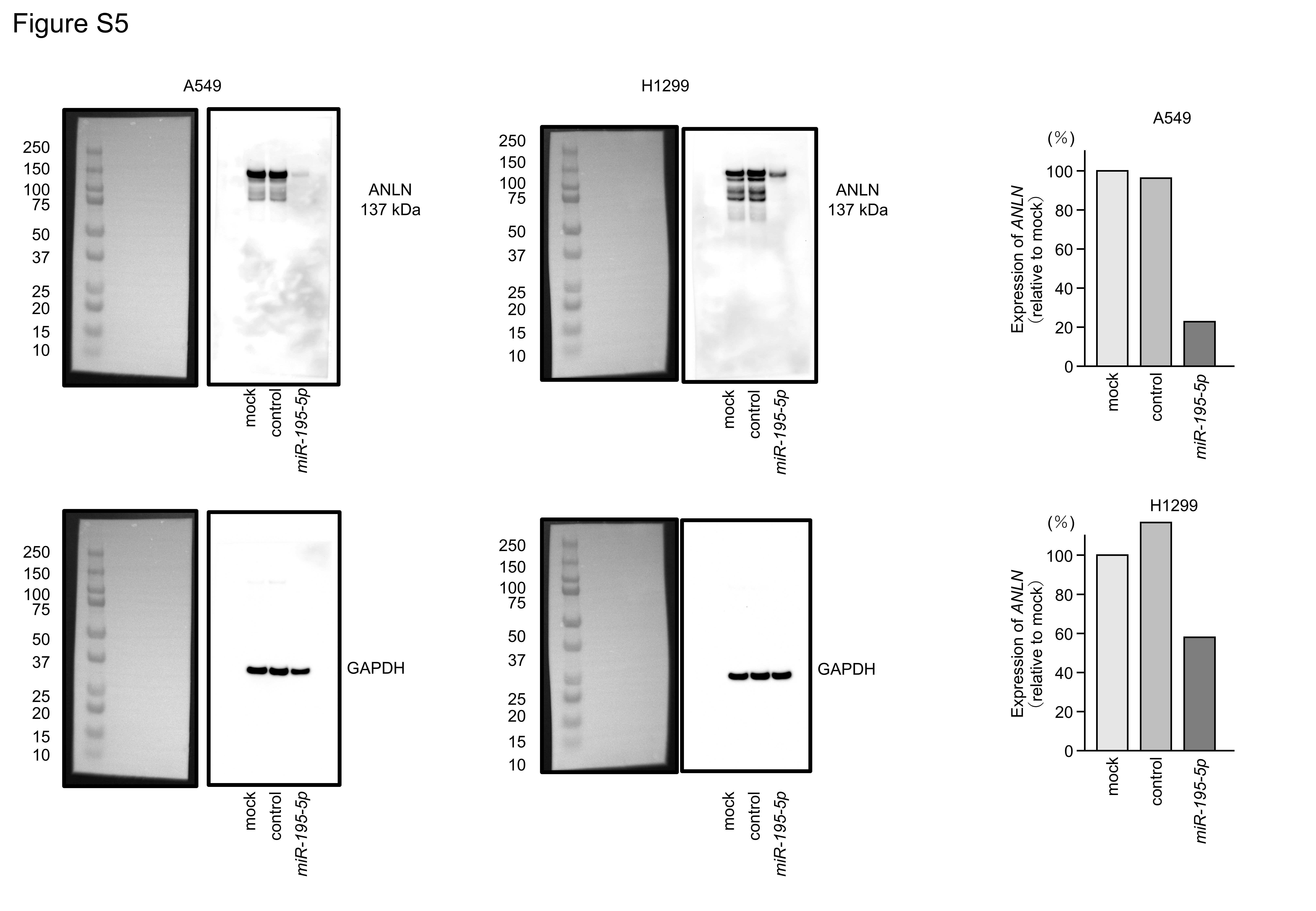

Supplement: Supplementary file 1 [file cancers-17-02348-s001.zip › cancers-3723267/Figure S5.tif]

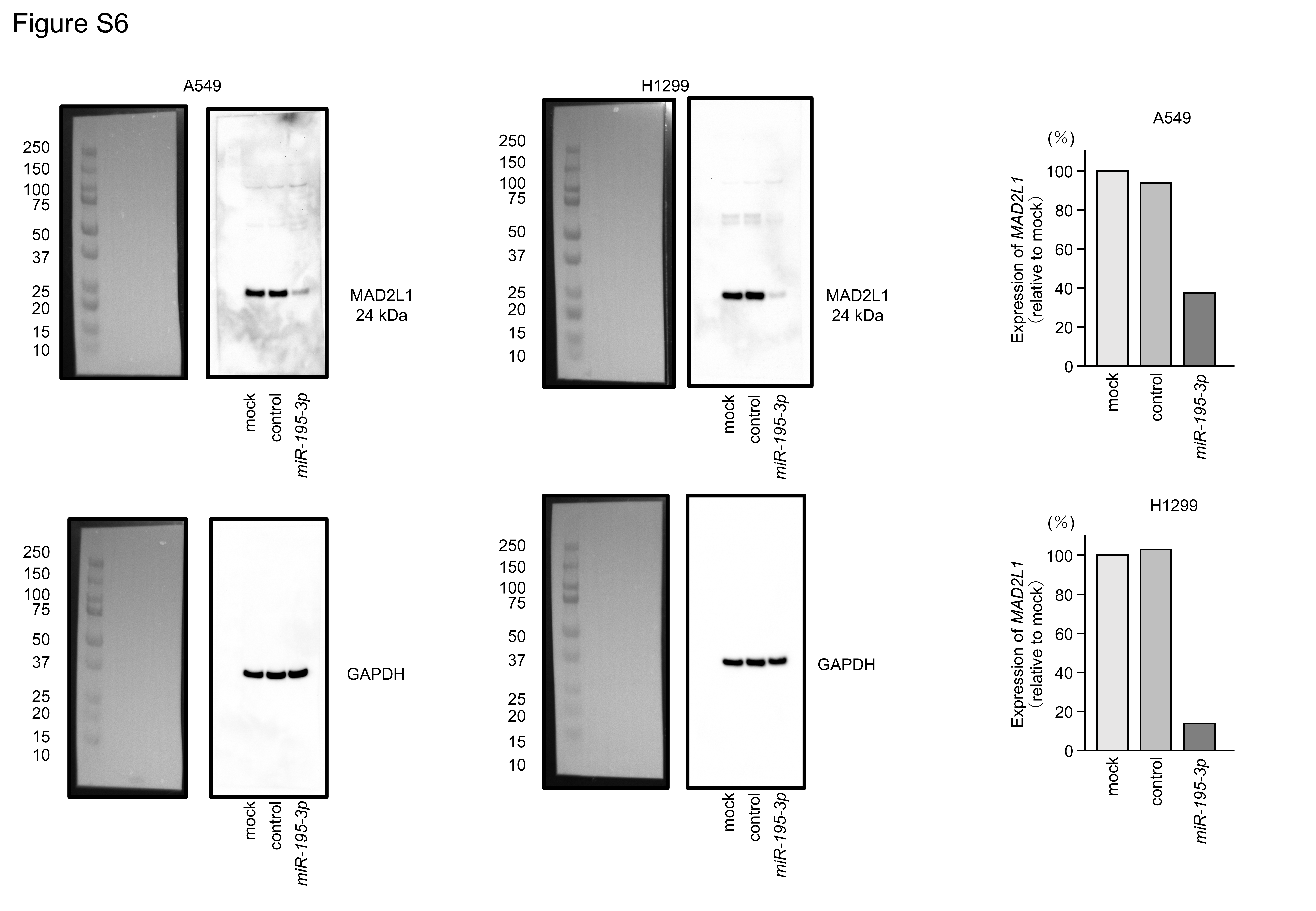

Supplement: Supplementary file 1 [file cancers-17-02348-s001.zip › cancers-3723267/Figure S6.tif]

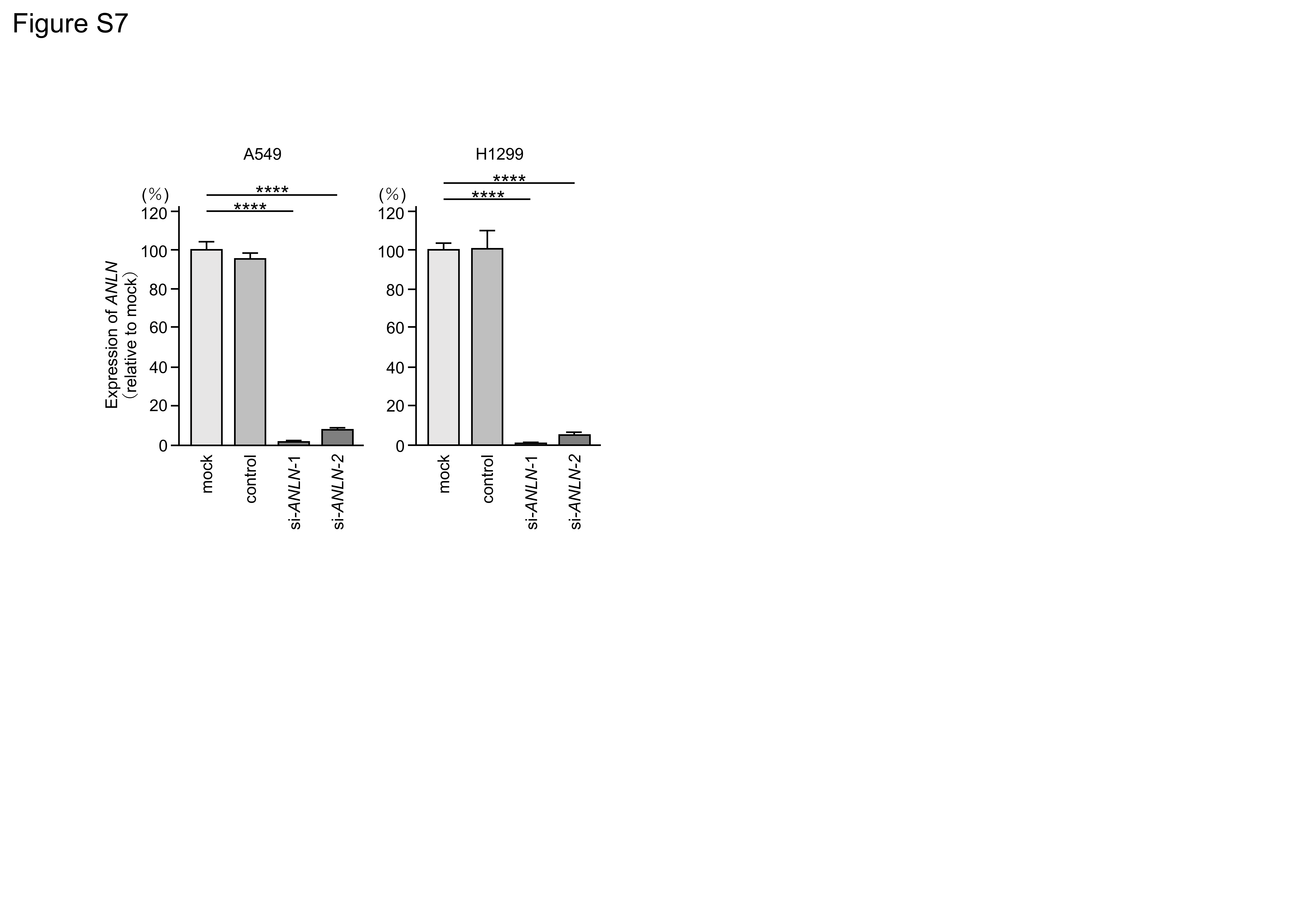

Supplement: Supplementary file 1 [file cancers-17-02348-s001.zip › cancers-3723267/Figure S7.tif]

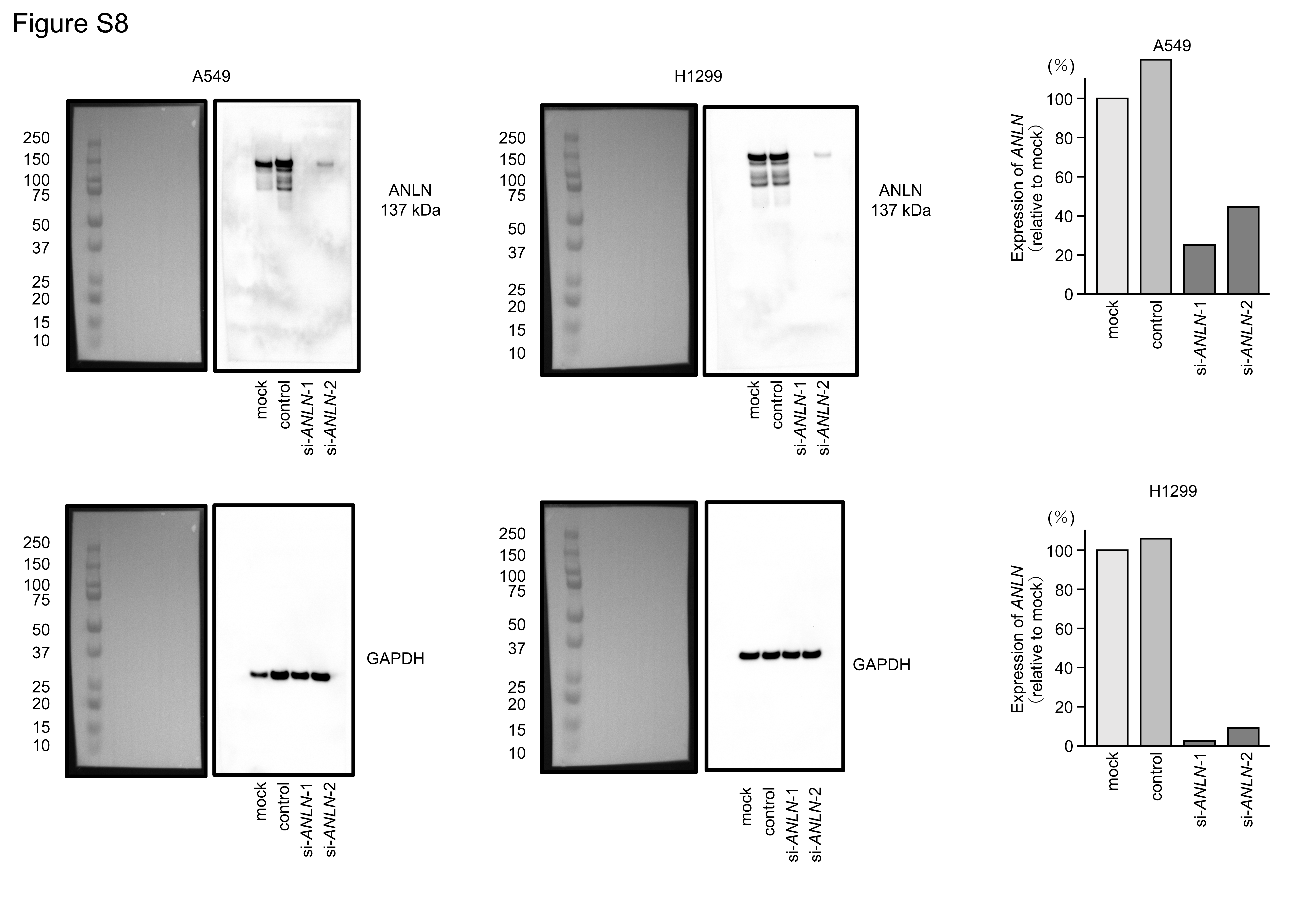

Supplement: Supplementary file 1 [file cancers-17-02348-s001.zip › cancers-3723267/Figure S8.tif]

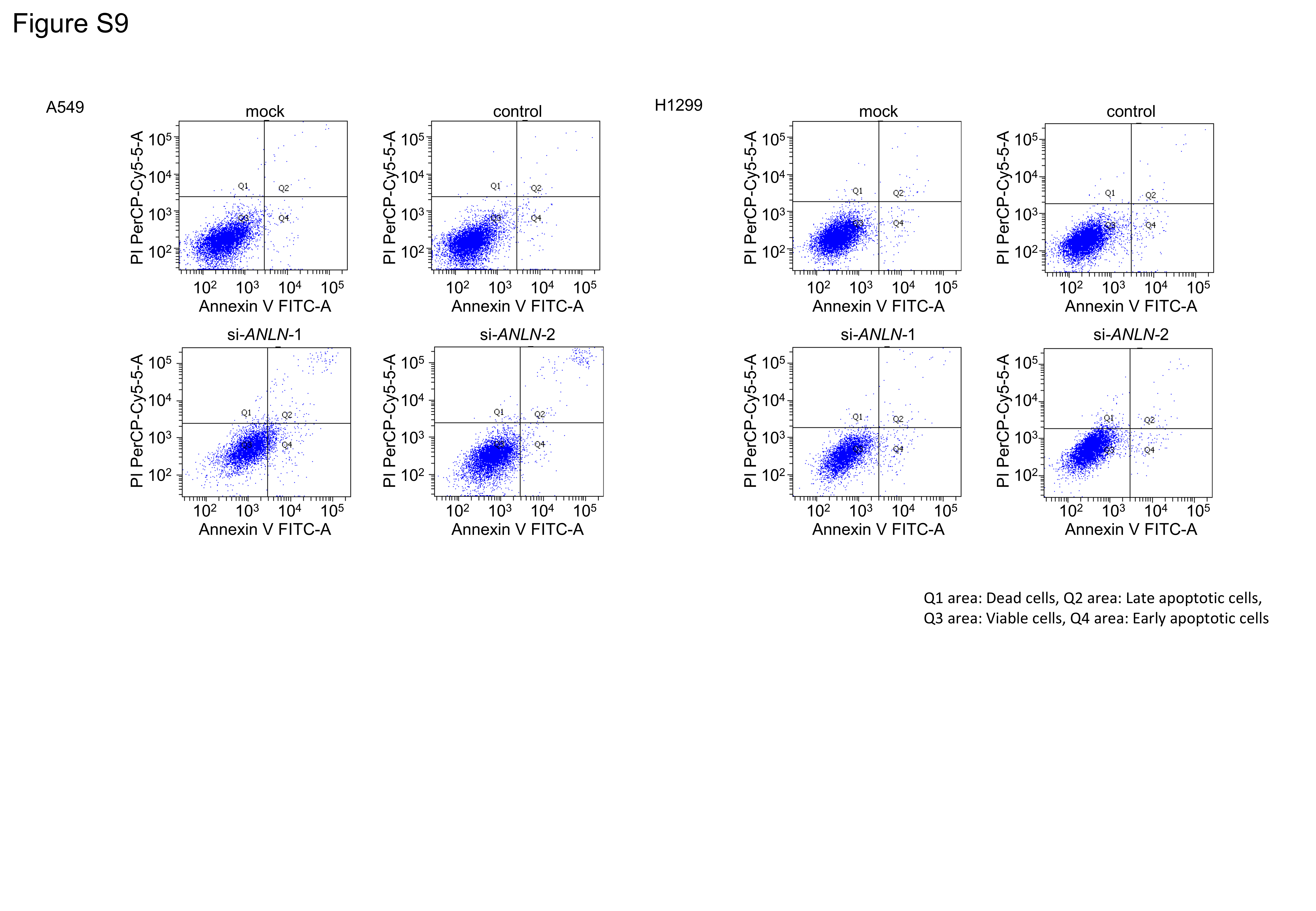

Supplement: Supplementary file 1 [file cancers-17-02348-s001.zip › cancers-3723267/Figure S9.tif]

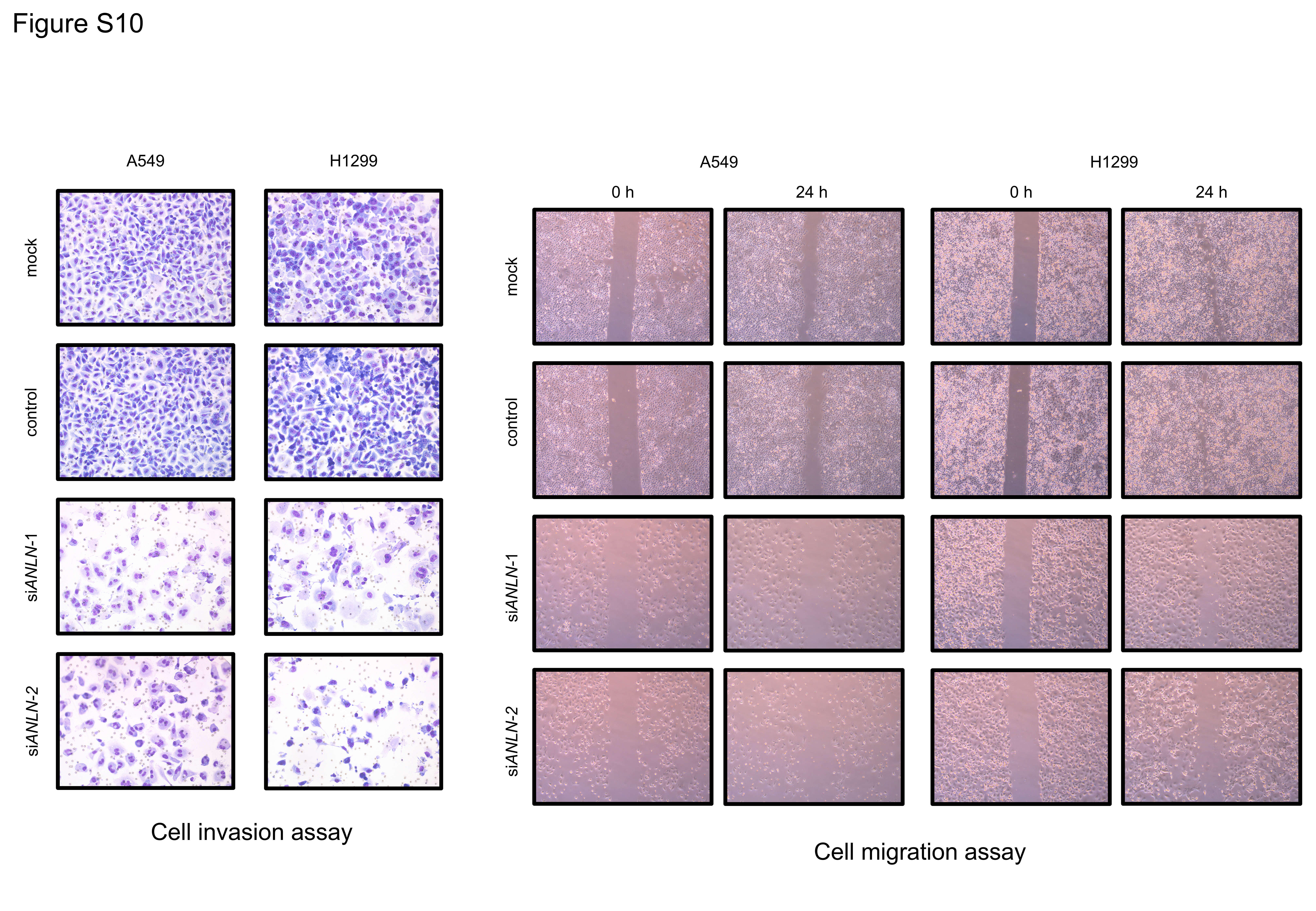

Supplement: Supplementary file 1 [file cancers-17-02348-s001.zip › cancers-3723267/Figure S10.tif]

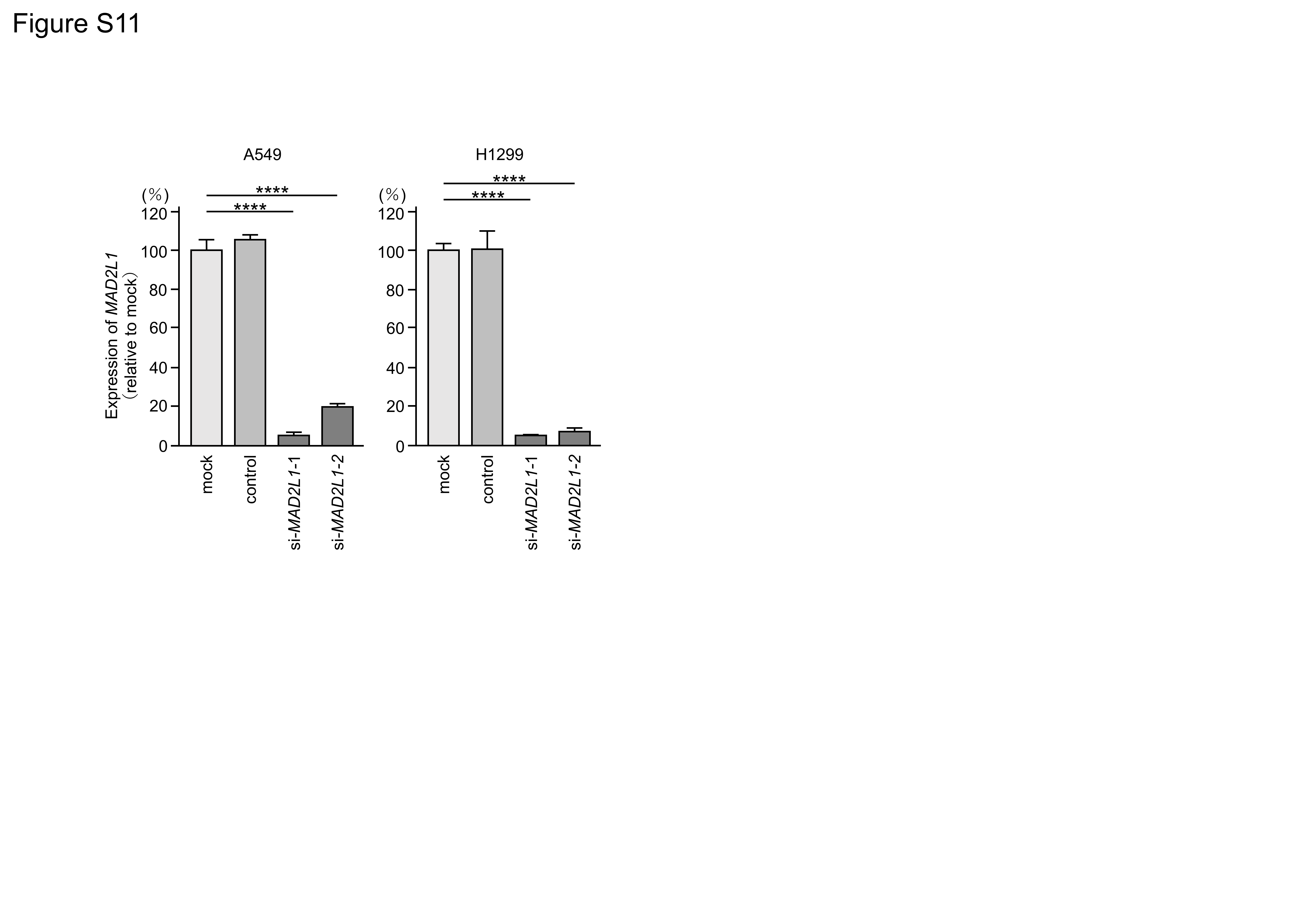

Supplement: Supplementary file 1 [file cancers-17-02348-s001.zip › cancers-3723267/Figure S11.tif]

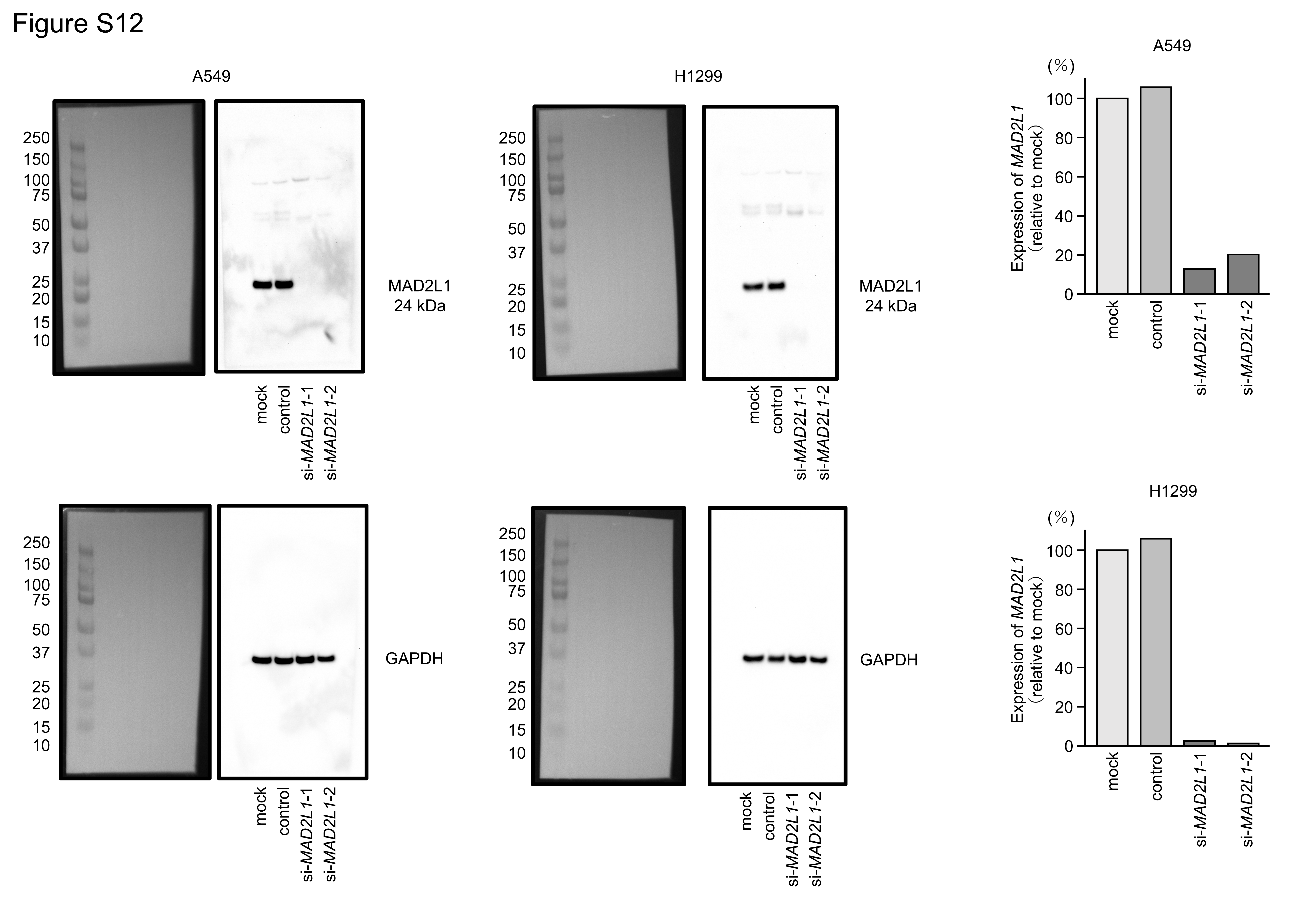

Supplement: Supplementary file 1 [file cancers-17-02348-s001.zip › cancers-3723267/Figure S12.tif]

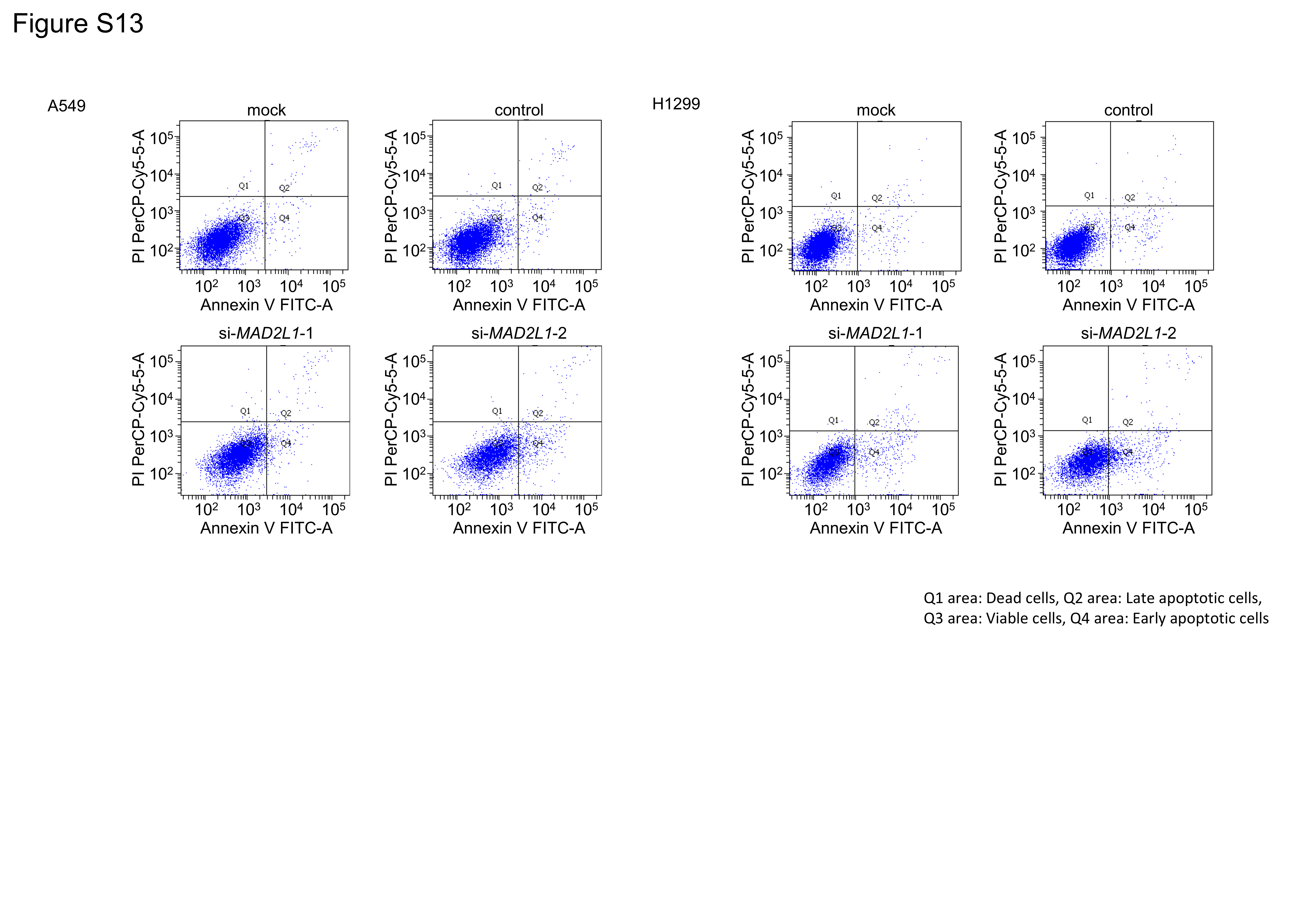

Supplement: Supplementary file 1 [file cancers-17-02348-s001.zip › cancers-3723267/Figure S13.tif]

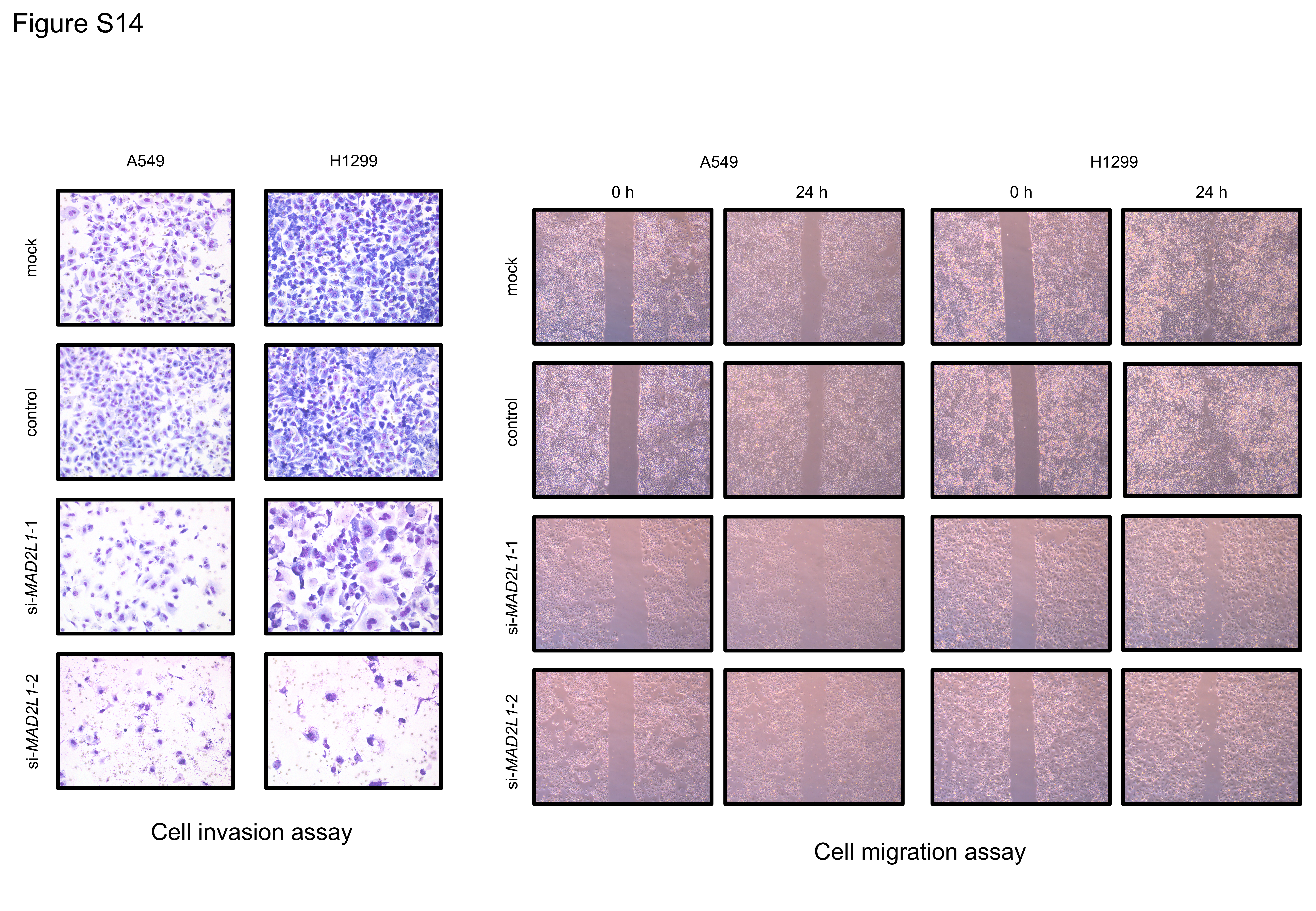

Supplement: Supplementary file 1 [file cancers-17-02348-s001.zip › cancers-3723267/Figure S14.tif]
